# Integrating bulk and single-cell RNA sequencing with GWAS reveals regulatory networks underpinning complex traits in beef cattle

**DOI:** 10.1186/s40104-026-01471-2

**Published:** 2026-07-28

**Authors:** Boyu Zhang, Shiyuan Qiu, Zhenwei Du, Hao Xiao, Yingxiao Su, Anyi Tang, Binwu Bao, Bo Zhu, Yan Chen, Xue Gao, Lingyang Xu, Zezhao Wang, Lupei Zhang, Huijiang Gao, Junya Li, Caihong Zheng

**Affiliations:** 1 State Key Laboratory of Animal Biotech Breeding, Institute of Animal Sciences, Chinese Academy of Agricultural Sciences, Haidian District, Yuanmingyuan West Road 2#, Beijing, 100193 China; 2 College of Animal Science and Technology, Ningxia University, Yinchuan, 750021 China

**Keywords:** Beef cattle, Complex trait, GWAS, Multi-transcriptomic data, scRNA-seq, Tissue and cell-type specificity

## Abstract

**Background:**

The genetic dissection of complex traits in livestock continues to pose a significant challenge in the field of animal genetics and breeding. Although traditional genome-wide association studies (GWAS) are capable of localizing genetic variants associated with specific traits, they are insufficient to elucidate the underlying physiological mechanisms. An integrated analysis of multi-trait GWAS and multi-transcriptomic data systematically identifies key tissues and cell types influencing complex traits in beef cattle and elucidates their genetic regulatory basis.

**Results:**

We systematically mapped tissue- and cell-type-specific regulatory architectures underlying 20 economically important traits in beef cattle. Tissue-level analyses revealed distinct trait-tissue associations: fatty acid traits, including C16:0 and C20:4, were enriched in liver; carcass traits, including marbling score and carcass weight, in renal cortex/medulla and longissimus dorsi muscle; meat-quality traits such as pH in cartilaginous tissues; and total fat content in bone marrow. At cellular resolution, analysis of eight trait-associated tissues identified 38 discrete cell types. Myofibers were significantly associated with most carcass traits, including rib-eye area and backfat thickness, whereas hepatocytes emerged as key regulators of fatty acid and meat-quality traits, such as C16:0 and crude protein content. Transcription factor analysis identified cell-type-specific regulators: TBX15, SOX6, and TCF12 in myofibers; FOXA2 and NR1H4 in hepatocytes; and IRF8 and IKZF1 in microglia. Notably, hepatocytes and microglia showed complementary, trait-specific association patterns: hepatocytes were enriched for C16:0 associated saturated fatty-acid metabolic pathways, while microglia were enriched for C16:1 and unsaturated fatty-acid–related pathways, suggesting potential cross-tissue coordination in lipid regulation.

**Conclusions:**

Our study links specific tissues and cell types to phenotypic variation in beef cattle and identifies core transcriptional regulators and pathways driving trait variation. These cell-resolved maps provide mechanistic insight into how genetic variation shapes economically important traits, offering a valuable resource for functional studies, cell-informed precision breeding strategies, and the design of large-scale molecular phenotyping.

**Supplementary Information:**

The online version contains supplementary material available at 10.1186/s40104-026-01471-2.

## Background

Genetic improvement of domesticated species, including beef cattle, requires a deeper understanding of how genomic variation shapes economically important traits such as carcass yield, meat quality, and fatty acid composition. Genome-wide association studies (GWAS) have identified hundreds of loci associated with these traits in beef cattle [1]. However, pinpointing the causal genes and elucidating the molecular mechanisms underlying these associations remains challenging, largely because many lead variants are in linkage disequilibrium (LD) with nearby causal variants and are located in noncoding regions of the genome [2–4].

Integrating GWAS with functional genomics resources—including molecular quantitative trait loci (molQTL) maps when genotype-matched molecular phenotypes are available—provides a powerful strategy to bridge this gap. By linking regulatory variation to trait-associated loci, molQTL studies can help identify putative causal genes, define tissue and cell-type relevance, and reveal the regulatory mechanisms that contribute to phenotypic variation. Large-scale efforts such as the Genotype-Tissue Expression (GTEx) project have systematically characterized genetic effects on the human transcriptome across diverse tissues, and linked regulatory variation to traits and diseases [5]. Building on this framework, FarmGTEx has extended regulatory mapping to livestock species by generating context-specific regulatory maps [6–8]. In cattle, the CattleGTEx project has linked gene expression across more than 20 tissues to 43 economically important traits, providing insights into their regulatory basis [9]. Recent advances in single-cell transcriptomics enable characterization of genetic regulatory mechanisms across diverse cell types and states [10–12]. Together with computational deconvolution, a comprehensive cattle single-cell atlas has enabled the generation of a cell type-resolved map of regulatory variants and advanced our understanding of the genetic and molecular architecture underlying cattle phenotypes [13, 14].

Despite these advances, key limitations persist. First, the functional interpretation of cattle GWAS signals remains constrained by the limited availability of high-quality, genotype-matched molecular phenotypes across tissues relevant to beef production. For many complex traits, the specific tissues and cell types through which genetic variation exerts its effects remain poorly defined [15]. This challenge is compounded by systematic genetic studies showing that complex traits often arise from coordinated regulatory programs spanning multiple tissues, with both shared and tissue-specific effects contributing to phenotypic variation [5, 16, 17]. Although the CattleGTEx framework represents a major step forward, its current implementation relies heavily on public datasets with variable data quality, small cohort sizes, heterogeneous breed composition, and batch effects, all of which limit the resolution and interpretability of livestock molQTL maps. In addition, the high cost of large-scale single-cell profiling makes rational prioritization of trait-relevant tissues a persistent challenge.

On the other hand, gene- and gene set-based association analyses have emerged as alternative frameworks for integrating GWAS with bulk or single-cell expression data [18]. By aggregating variant effects into gene-level statistics and testing their enrichment in tissue-specific or cell-type-specific expression profiles, regulatory annotations, or co-expression modules, these approaches improve causal gene prioritization and help infer trait-associated tissues and cell types [18, 19]. The resulting trait-associated gene sets provide a useful substrate for downstream analyses, including inference of trait-relevant tissues and cell types and delineation of biological pathways that contribute to phenotypic variation [15, 20–22].

In this study, we systematically delineate the genetic regulatory architecture underlying economically important traits in beef cattle by integrating multi-trait GWAS with multi-scale transcriptomic resources, including multi-tissue bulk expression and single-cell RNA-seq. Using GWAS summary statistics in combination with expression profiles from 47 tissues, we first prioritized tissues most relevant to each trait. We then generated single-cell RNA-seq data from a subset of these tissues—including liver, longissimus dorsi muscle, medulla oblongata, renal cortex, renal medulla, rumen, rib cartilage, and bone marrow—and resolved 38 distinct cell types. By integrating these single-cell profiles with GWAS signals, we identified trait-associated cell types and the cellular contexts in which genetic variation is most likely to exert its effects. We further used fatty-acid traits, including C16:0 and C16:1, to illustrate distinct cell-type enrichment patterns. Complementary gene-set, functional enrichment, and transcriptional network analyses further revealed candidate regulatory circuits and biological pathways that may contribute to trait variation, both within specific tissues and through coordinated cross-tissue mechanisms. Together, these results provide a high-resolution view of the cellular mechanisms that link genetic variation to phenotypic diversity in beef cattle and establish a framework for future mechanistic and breeding-oriented investigations.

## Materials and methods

### Genotypes, imputation and quality control

We collected phenotypic and genotypic data from 1,788 Huaxi cattle (a specialized Chinese beef breed developed through crossbreeding and selection programs that utilized the Simmental, Mongolian, Sanhe, and Charolais breeds) for 10 traits spanning carcass, meat-quality, and fatty-acid categories (Table S1). All animals were genotyped using the Illumina BovineHD 770 K BeadChip. Whole-genome resequencing data from 94 Huaxi cattle were used as a within-population reference panel to impute chip-based genotypes to sequence-level variants using Beagle v5.4 [23].

Genotype quality control was performed using PLINK v1.90 [24]. SNPs and individuals with missingness > 10% were removed, and variants with MAF < 0.05 were excluded. Imputed variants were filtered using Beagle’s dosage R-squared metric (DR^2^) reported in the VCF INFO field; variants with DR^2^ < 0.8 were removed. Dosage was defined as the expected alternate-allele count (0–2) for each of the 1,788 imputed individuals, computed from Beagle posterior genotype probabilities. For the resequenced reference-panel VCF, additional filters using VCFtools v0.1.17 [25] removed variants with missingness > 50%, MAC < 3, QUAL < 30, or genotype depth < 3. After QC, we retained 10,095,765 high-confidence autosomal variants for downstream analyses, with a mean imputation DR^2^ of 0.92 across retained variants.

### Genome-wide association analysis

Prior to conducting GWAS, phenotypes were adjusted for year, sex, age and the first two genetic principal components (calculated in PLINK) as covariates. GWAS were then performed using GEMMA 0.98.5 [26] under a linear mixed model (LMM) framework.

### GWAS summary statistics

In this study, we analyzed GWAS summary statistics from two sources. First, summary-level association results for 10 agronomic traits in Chinese Simmental cattle were obtained from a previously published study [27]. These Chinese Simmental cattle serve as the paternal line in the breeding scheme of the Huaxi cattle population. Second, we generated GWAS summary statistics for 10 carcass, meat-quality, and fatty-acid traits in Huaxi cattle, as described in the sections “Genotypes, imputation and quality control” and “Genome-wide association analysis”. For each trait, single-SNP association tests were performed under a linear mixed model framework, and genome-wide summary statistics (SNP ID, chromosome, position, effect size, standard error and *P* value) were used in downstream integrative analyses.

### SNP annotation and gene mapping

Genome-wide significant SNPs (Table S2) were annotated using bedtools v2.31.1 [28]. Significant SNPs were first converted to BED format (chromosome, start = position − 1, end = position), while retaining SNP IDs, rsIDs, and *P* values as additional columns. Exon and coding sequence (CDS) regions were extracted from the reference GTF file to generate BED files of functional genomic features. We then used the intersect subcommand of bedtools to identify overlaps between significant SNPs and gene features (e.g., exons and CDS). To capture potential regulatory target genes, we further used the window subcommand of bedtools to assign genes within ± 50 kb of each SNP. Finally, annotations from the intersect- and window-based approaches were combined to generate, for each SNP, a list of candidate genes for downstream analyses (Table S3). The ± 50 kb window was chosen to capture proximal regulatory variants, in line with commonly used settings in gene-based analyses.

### Gene expression pattern across tissues

Tissue expression data were generated from three Chinese Simmental bulls previously collected by our research team at Shayang Hanjiang Cattle Development Co., Ltd. (Hubei Province, China). After weaning, all calves were raised under identical feeding and management conditions until 2 years of age. In total, 132 tissue samples representing major organ systems (e.g., digestive and immune systems) were collected and grouped into 47 tissue types based on biological replicates. Details of the samples are provided in Table S4. All samples were processed using standard RNA-seq library preparation and sequencing procedures to generate transcriptome profiles [29]. To characterize gene expression patterns across the 47 bovine tissue types, we retained genes expressed in at least two biological replicates per tissue and calculated the mean expression level across replicates. Gene expression values were then log2-transformed. We next calculated the variance of each gene across all samples and selected the 500 most variable genes for principal component analysis (PCA). The resulting principal components were used to visualize tissue clustering patterns, and hierarchical clustering was generated using the R package ggdendro v0.2.0.

### Tissue-specific gene selection

A gene-by-tissue expression matrix was constructed using FPKM values, with genes as rows and tissues as columns. Genes with a maximum FPKM < 0.1 across all tissues were removed to reduce low-expression background noise. For the remaining genes, FPKM values were transformed as $\log_2(\mathrm{FPKM}+1)$, and standardized to Z-scores within each gene to make expression levels comparable across tissues. For each tissue, that tissue was in turn treated as the “target” group (*n*_1_ ≥ 1), and all other tissues were combined as the “reference” group (*n*_2_ > 1). Expression differences between the target and reference groups were quantified using the two-sample *t*-statistic for independent groups, as defined by the following formula:

$$\begin{array}{c}{t}_{i}=\frac{{\overline{x}}_{t}-{\overline{x}}_{o}}{\sqrt{\frac{{v}_{t}}{{n}_{t}}+\frac{{v}_{o}}{{n}_{o}}}}\end{array}$$

where *i* indexes genes; ${\overline{x}}_{t}$ and ${\overline{x}}_{o}$ represent the mean transformed expression levels of gene *i* in the target tissue and the reference group (all other tissues), respectively; ${v}_{t}$ and ${v}_{o}$ are the corresponding sample variances; and ${n}_{t}$ and ${n}_{o}$ are the numbers of samples in the target and reference groups, respectively. All tissues used for tissue-specific gene selection had at least two biological replicates (*n*_1_ ≥ 2); therefore, *t*-statistics were computed only for tissues with *n* ≥ 2 replicates. For each tissue, genes with *t*-statistics ranking in the top 10% were defined as tissue-enriched genes (top 10%), following a commonly used operational definition in expression-based tissue/cell-type enrichment frameworks [30]. To further identify genes with highly restricted expression, the Phi coefficient (*ϕ*) was calculated as follows:

$$\begin{array}{c}\phi =\frac{{n}_{11}{n}_{00}-{n}_{10}{n}_{01}}{\sqrt{{n}_{1\bullet }{n}_{0\bullet }{n}_{\bullet 0}{n}_{\bullet 1}}}\end{array}$$

where $n_{11,}n_{00,}n_{10,\;}\mathrm{and}\;n_{01}$ are the four cell counts in a 2 × 2 table defined by tissue membership (target vs. other) and whether a gene is classified as “highly expressed” in a sample (high vs. not high); $n_{1\bullet,}n_{0\bullet},n_{\bullet0,}n_{\bullet1}$ are the corresponding marginal totals. The Phi coefficient measures the strength of association between these two binary variables. Genes with |*ϕ*| ≥ 0.95 were considered highly specific to the target tissue and were designated as “exclusive tissue-specific genes” (Table S5). The top 10% genes capture broadly tissue-enriched genes, whereas genes with |*ϕ*| ≥ 0.95 represent a more stringent subset with nearly exclusive expression in the target tissue.

### Enrichment analysis of tissue-specific genes and complex traits

Two complementary methods were employed to infer trait-associated tissues: DESE [31] and MAGMA [19]. Both approaches require GWAS summary statistics and multi-tissue gene expression profiles, with data sources described above.

DESE, implemented in KGGSum v1.0, was performed using the KGGSEE DESE (driver-tissue inference) framework, which simultaneously conducts phenotype–tissue association tests and conditional gene-based association tests using GWAS summary statistics and multi-tissue expression profiles. SNPs were mapped to genes using a ± 50 kb window, and gene coordinates were provided via a customized gene model database in BED format. Here, this BED file refers to a BED-formatted gene annotation file containing gene coordinates for DESE, whereas the BED format mentioned above refers to the BED-formatted representation of significant SNPs. We set gene-p-cut = 0.05 as the gene-level inclusion threshold in the DESE workflow, and significance was controlled using Benjamini–Hochberg FDR (*q* < 0.05). To improve interpretability, tissue-selective expression scores were supplied via --gene-score-file (GeneScore), and DESE tested whether phenotype-associated genes tend to show higher tissue-selective scores in each tissue, reporting tissue enrichment statistics (Z-scores).

As a secondary analysis, we applied MAGMA v1.10 in R to evaluate the association between tissue-specific gene expression and GWAS signals. SNPs were annotated to genes by including the gene body and ±100 kb flanking regions (--annotate window = 100, in kb). Gene-level association statistics were computed using GWAS summary statistics (--pval, specifying the corresponding SNP and *P*-value columns) and LD estimated from a matched cattle reference panel in PLINK binary format (--bfile) derived from the 1000 Bull Genomes Project. For each tissue, we constructed a tissue-specific gene set by selecting the top 10% genes ranked by tissue-specific expression (as defined in the Tissue-specific gene selection section) and performed competitive gene-set analysis in MAGMA using --gene-results (gene-level results) and --set-annot (gene-set annotation). We did not impose an additional per-gene SNP cap beyond MAGMA’s default handling; multiple testing across tissues/gene sets was controlled using Benjamini–Hochberg FDR (*q* < 0.05).

For functional interpretation, KEGG over-representation analysis was performed using trait-associated genes from MAGMA gene-level results (BH-FDR *q* < 0.05), with all genes tested in MAGMA as the background. KEGG pathway–gene mappings for *Bos taurus* were retrieved using the KEGGREST R package and converted to the gene IDs used in this study prior to enrichment testing.

To prioritize tissues for downstream single-cell profiling, we integrated MAGMA and DESE results by ranking tissues within each method and selecting those that appeared among the top eight for a given trait in both analyses.

### Preprocessing of snRNA-seq data and scRNA-seq

This study analyzed single-nucleus and single-cell RNA sequencing (snRNA-seq and scRNA-seq) data from nine samples across eight tissues of Huaxi cattle, generated from two sources. Sample information and processing procedures are summarized in Table S6. The bovine reference genome (ARS-UCD1.2) and corresponding gene annotation file (GTF, version 108) were obtained from the Ensembl database (Release 108) [32]. The genomic FASTA and GTF files were then used to build a Cell Ranger–compatible reference transcriptome with Cell Ranger v9.0.1. Raw snRNA-seq and scRNA-seq reads were aligned to the bovine reference genome, and barcode assignment and unique molecular identifier (UMI) counting were performed using the standard Cell Ranger workflow, resulting in a gene-by-cell expression matrix for each sample. To ensure data quality, potential doublets were identified and removed using DoubletFinder v2.0.6 [33]. Cells expressing fewer than 200 genes and cells with > 10% of UMIs mapped to mitochondrial genes were excluded. In addition, an adaptive filtering strategy based on the median absolute deviation (MAD) of gene and UMI counts within each cluster was applied to remove outlier cells. During gene filtering, genes detected in fewer than three cells were removed from downstream analyses.

### Identification of cell clusters

Unsupervised clustering was performed using Seurat v5.3.0 [34]. Libraries from all samples were merged into a single Seurat object and subjected to normalization and scaling. Variable genes were identified using the FindVariableFeatures function with selection.method = "vst" and nfeatures = 2,000. PCA was then performed on the merged object, and the top 30 principal components were retained. Batch effects across libraries/samples (defined by Seurat metadata field orig.ident) were corrected using RPCAIntegration implemented in Seurat v5 IntegrateLayer. PCA was used as the original dimensional reduction input (orig.reduction = "pca") to obtain an integrated low-dimensional representation. In this integrated space, the top 30 integration components were used to construct a shared nearest-neighbour graph, and cell clusters were identified using the FindClusters function with a resolution of 0.9 and visualized using UMAP. Each cluster was manually annotated based on well-established marker genes reported in the literature.

### Cell cycle index estimation

To characterize cell-state dynamics, cell-cycle phase scores were computed using the CellCycleScoring function in Seurat [34]. Based on canonical S and G2/M gene sets, cells were assigned to one of three cell-cycle phases: G0/G1, S or G2/M.

### Enrichment analysis between cell types and complex traits

scPagwas v1.3.0 [35] was employed to perform enrichment analysis between cell types and complex traits. scPagwas applies a polygenic regression model to prioritize trait-associated genes and identifies trait-relevant cell types by integrating pathway activity scores derived from scRNA-seq data with GWAS summary statistics.

To improve the robustness of the analysis, we retrieved 319 human KEGG pathways from the KEGG database and removed duplicate entries. Pathway genes were then mapped to cattle using Ensembl Compara orthology annotations; one-to-one orthologs were retained directly, whereas one-to-many and many-to-many relationships were resolved by selecting the best-supported ortholog.

We ran scPagwas with cattle-specific LD inputs (block_annotation and chromosome-wise chrom_ld) and set min.pathway.size = 70, iters_singlecell = 10, and singlecell = TRUE. The Boot_evaluate function was used to identify significantly trait-associated cell types and to calculate the trait relevance score (TRS). The scGet_PCC function was applied to rank genes based on their Pearson correlation coefficient (PCC) values, and, for each cell type, the top 100 genes by PCC were defined as trait-associated genes. In addition, the scPagwas_perform_score was used to estimate pathway activity, and the significance of active pathways in each cell type was determined using singular value decomposition. Enrichment analysis of trait-associated genes and active pathway genes across cell types were mainly assessed using one-tailed Fisher’s exact tests (alternative = “greater”).

Module scores for gene sets were calculated using Seurat’s AddModuleScore, which computes relative per-cell scores by subtracting the aggregated expression of expression-matched control gene sets (randomly sampled from bins of similar average expression) from the average expression of the target gene set.

### Functional enrichment analysis and gene-set enrichment validation

Gene Ontology (GO) and KEGG pathway enrichment analyses were performed using clusterProfiler v4.17.0 [36]. The enrichGO and enrichKEGG functions were used to identify enriched GO terms and KEGG pathways for selected genes, with the org.Bt.eg.db annotation package and the bovine KEGG organism code (“bta”), respectively, and a significance threshold of pvalueCutoff = 0.05. Differentially expressed genes for each cell type were identified using Seurat v5.3.0 [34], and a ranked gene list was constructed based on a composite metric of log_2_FC and adjusted *P*-value. Bovine KEGG and GO gene sets were obtained from the Molecular Signatures Database (MSigDB) via the msigdbr v7.5.1 package. The gene set enrichment analysis (GSEA) functions were then applied to calculate pathway scores for each cell-type-specific gene set across pathways.

To assess whether one gene set was significantly enriched within another, we performed a hypergeometric test based on the overlap between the two sets [37]. Let *N* denote the total number of background genes, *n* the number of genes in gene set *A*, *K* the number of genes in gene set *B*, and *k* the number of genes shared by both sets. The probability of observing at least *k* overlapping genes by chance was computed using the hypergeometric distribution:

$$P=1-{P}_{hypergeometric}(k-1,K,N-K,n)$$

All genes were restricted to the background gene list before calculation. To validate the robustness of the enrichment, Fisher’s exact test (one-tailed, alternative = “greater”) was also applied to the same contingency table. Both analyses were implemented in R using the base phyper() and fisher.test() functions.

### Gene regulatory network analysis

Gene regulatory network analysis was performed using the pySCENIC pipeline. First, the integrated single-cell objects were imported into Seurat, and the gene expression matrix from the RNA assay was extracted. Preliminary gene filtering was performed by retaining genes expressed in at least 1% of cells and with a total expression count of at least 3 × 1% × the total number of cells (equivalent to 0.03 × the total cell count). The filtered gene-by-cell expression matrix was then exported for downstream analysis. Subsequently, the function “orthologs()” from the babelgene R package (v22.9) was used to convert bovine genes into their human homologues, which were used for regulatory network inference and enrichment analysis with pySCENIC v.0.12.1.

Gene regulatory network inference was performed using the Python package pySCENIC v.0.12.1 [38] with default parameters. Co-expression modules were identified via the GRNBoost2 algorithm, using the human TFs list as reference. Next, *cis*-regulatory modules were refined by retaining genes directly targeted by TFs based on motif enrichment within 10 kb of the transcription start site, using cisTarget databases for Homo sapiens (hg38, RefSeq r80) from the SCENIC + resource and the gene-based cistarget databases provided by the Aerts lab. Regulon activity scores were quantified as the area under the recovery curve using AUCell. To identify key transcriptional regulators defining each cell identity, regulon specificity scores (RSS) [39] were calculated to assess the association between regulon activity and specific cell types.

### Statistical analysis

A range of statistical methods, including linear mixed models, multiple-testing correction procedures, non-parametric tests and hypergeometric tests, were applied to ensure the robustness of the results. Unless otherwise specified, statistical analyses and data visualization were performed in R (version 4.5.1). Detailed descriptions of the statistical models, parameters and significance thresholds used in each analysis are provided in the corresponding methodological sections.

## Results

### GWAS of carcass, meat-quality, and fatty-acid traits

**Fig. 1:**
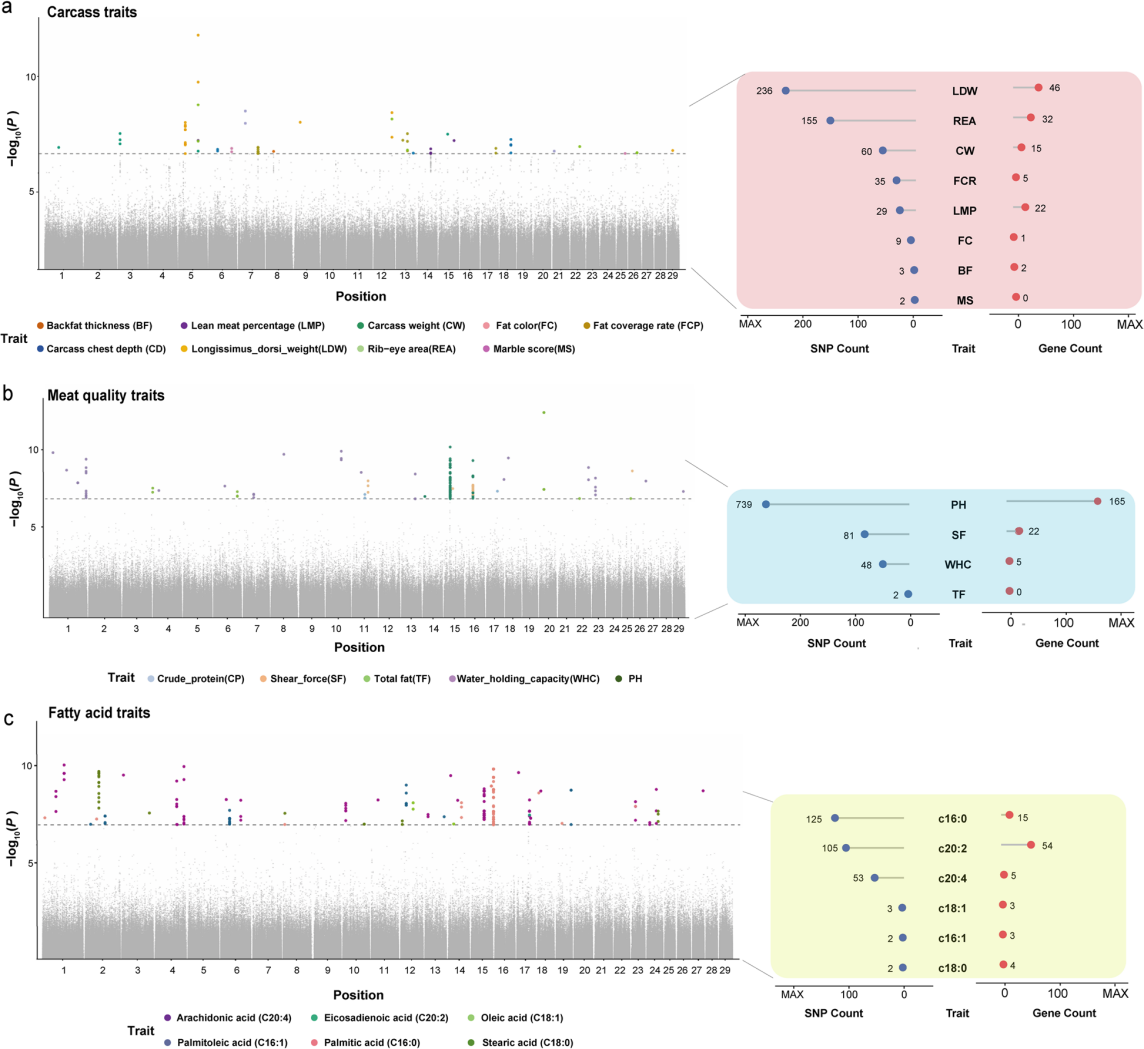
Genome-wide association studies for agronomic traits in beef cattle. **a**–**c** Manhattan plots of GWAS results for carcass traits (**a**), meat-quality traits (**b**), and fatty-acid traits (**c**). The horizontal dashed line indicates the genome-wide significance threshold (*P* = 1 × 10^−7^). Numbers on the right of each panel denote, for each trait group, the total number of significant SNPs and the number of genes annotated within 50 kb upstream or downstream of these loci

As a first step toward linking economically important traits to relevant cell types, we performed GWAS for 10 traits in Huaxi cattle using imputed sequence-level variants. In total, we analyzed 20 traits, including 10 traits generated in Huaxi cattle and 10 traits obtained from published Chinese Simmental GWAS summary statistics, spanning carcass, meat-quality, and fatty-acid categories (Fig. 1 and Table S1). Across these traits, we identified 1,689 unique genome-wide significant variants associated with at least one trait (Fig. 1 and Table S2). The number of significant variants per trait ranged from 2 for marbling score to 739 for meat pH (Fig. 1). Quantile–quantile (Q–Q) plots showed that most observed *P* values followed the expected null distribution, with modest tail inflation consistent with true association signals (Fig. S1 and Table S7).

Annotation of significant loci yielded 399 nearby genes across the 20 traits (Fig. 1 and Table S3). Meat pH exhibited the largest number of annotated genes (*n* = 165; Table S3). For C16:0 content, two candidate genes on chromosome 28, *PLA2G12B* and *ATG2B*, were identified within associated loci (Table S3). Both genes have previously been implicated in lipid-related biological processes [40, 41], supporting their potential relevance to fatty-acid variation in beef cattle.

### Gene expression across tissues

**Fig. 2:**
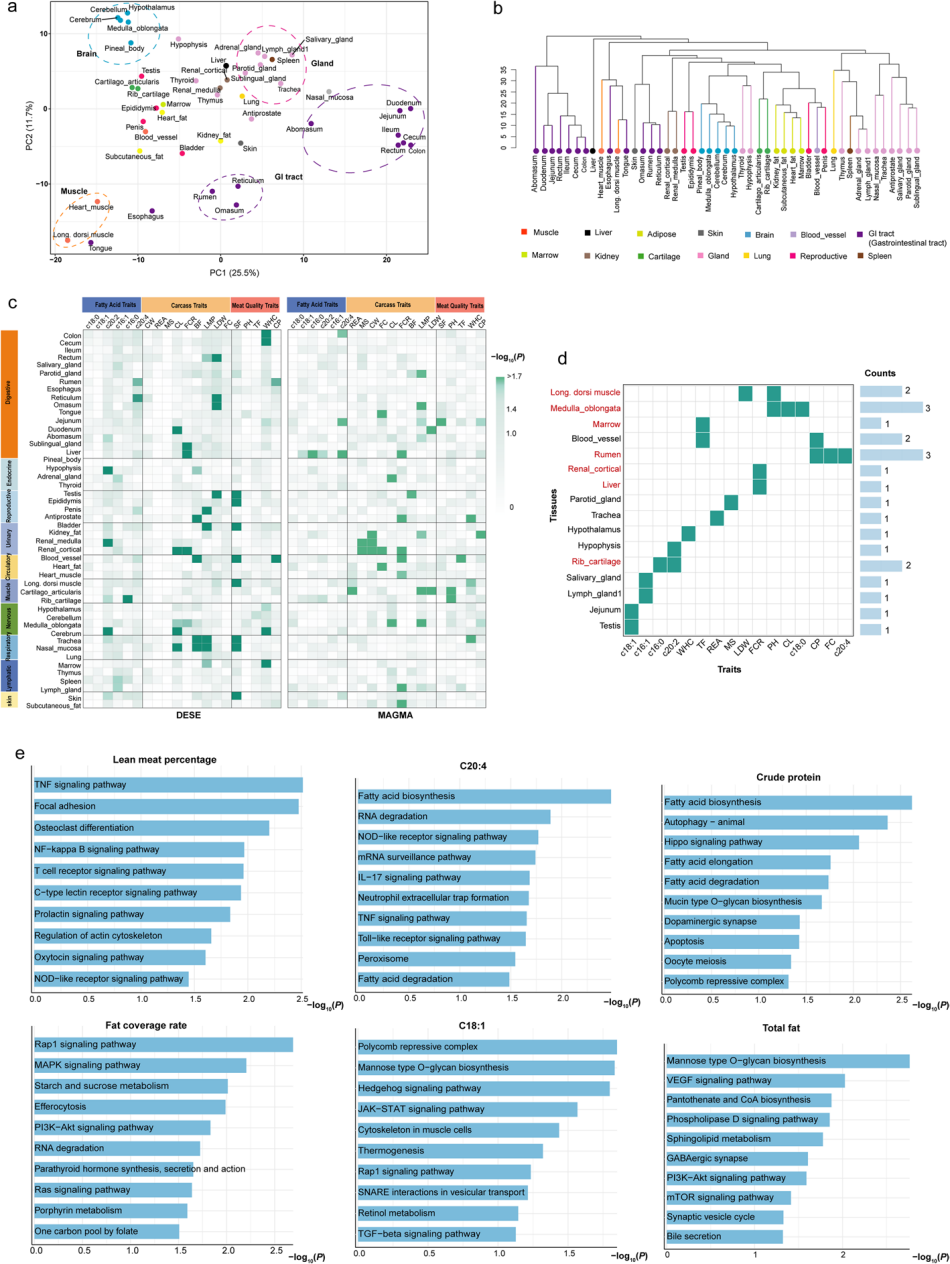
Enrichment analysis of complex trait signals across tissues. **a** PCA of normalized gene expression [log_2_(FPKM +1)] across 47 tissues. Each point represents one sample, colored by tissue group. **b** Hierarchical clustering dendrogram of the same tissues based on gene expression, showing that central nervous system, muscle and digestive tissues form distinct clusters. **c** Heat maps of trait–tissue associations inferred using DESE (left) and MAGMA (right). Color intensity indicates the strength of association between each of the 20 agronomic traits and tissue-specific gene expression. **d** Overlap of the top eight trait-associated tissues identified by each method. Bar length indicates, across all traits, the frequency with which each tissue appears among the top-ranked tissues. Tissues highlighted in red were selected for subsequent scRNA-seq based on overlap frequency and association patterns in (**c**). **e** KEGG pathway enrichment of genetic signals for 20 traits using MAGMA. Bar plots show enrichment significance for pathways associated with carcass, meat-quality and fatty-acid traits, with two representative traits displayed for each trait category. Long. dorsi muscle = longissimus dorsi muscle

To identify key regulatory tissues associated with agronomic traits, we first carried out a systematic analysis of bovine tissue transcriptomic profiles. PCA was performed on normalized expression levels of genes expressed in at least two biological replicates. The PCA revealed that tissues with similar physiological functions clustered closely in the expression space (Fig. 2a), consistent with previous reports [29]. We next applied hierarchical clustering using the average-linkage method and visualized the results as a dendrogram (Fig. 2b). The clustering pattern strongly mirrored the PCA structure, further supporting the notion that functionally related tissues share conserved co-expression modules. Specifically: (1) central nervous system tissues (including the medulla oblongata, hypothalamus, pineal gland, cerebrum and cerebellum) formed a tight cluster; (2) muscle tissues (myocardium and longissimus dorsi) were grouped together; and (3) digestive system tissues were partitioned into two main branches corresponding to the forestomach, abomasum and intestinal tract. Together, these results suggest that, in our dataset, inter-tissue variation in gene expression tracks functional specialization.

### Bulk transcriptome-based prioritization of trait-relevant tissues

To link trait-associated genetic signals with relevant tissues, we integrated GWAS results with bulk tissue expression profiles using MAGMA [19] and DESE [31]. In the MAGMA analysis, we tested the enrichment of the top 10% most specifically expressed genes in each tissue (Table S5) for trait-associated genetic signals (Fig. 2c). We observed that GWAS signals for several fatty-acid traits (e.g., C16:0 and C20:4) were significantly enriched in liver-expressed (tissue-enriched) genes, consistent with the liver’s central role in lipid metabolism [42]. Carcass traits such as marbling score (MS) and carcass weight (CW) showed significant enrichment of GWAS association signals among kidney cortex- and medulla-enriched gene sets, whereas GWAS signals for meat-quality traits such as pH were enriched among cartilage-enriched genes, and GWAS signals for total fat content (TF) were enriched among vascular tissue–enriched genes (Fig. 2c). Previous studies have highlighted the importance of vasculature in adipogenesis, whereby blood vessels not only supply oxygen and nutrients to adipose tissue but also promote preadipocytes differentiation through signaling molecules such as VEGF [43]. The kidneys are the main site for vitamin D activation, and 1,25(OH)_2_-vitamin D plays a key role in muscle anabolism [44]. Using DESE, we further observed that GWAS signals for C20:2 were enriched in brain-related tissue-selective genes (e.g., medulla oblongata and cerebrum), GWAS signals for longissimus dorsi weight (LDW) were enriched among testis-, stomach-, and rectum-enriched gene sets, and GWAS signals for crude protein content (CP) were enriched among vascular tissue- and rumen-enriched genes (Fig. 2c).

To prioritize tissues for subsequent single-cell transcriptomic analysis, we integrated the enrichment rankings from both MAGMA and DESE, and selected tissues that appeared within the top eight for each trait in both analyses (Fig. 2d). This integrative approach revealed that C20:4, fat color (FC), and CP were all associated with the rumen. LDW was enriched in longissimus dorsi muscle, as expected. TF, by contrast, showed co-enrichment in vascular tissue and bone marrow.

To further assess the biological relevance of the identified trait-associated genes, we performed KEGG pathway analysis. The enriched pathways were broadly consistent with the biological basis of the corresponding carcass, meat-quality, and fatty-acid traits (Fig. 2e). Among carcass traits, lean meat percentage (LMP) was significantly enriched in muscle-development–related pathways, including Focal adhesion [45], NF-kappa B signaling pathway [46] and TNF signaling pathway [47]. Fat coverage rate (FCR) was enriched in the PI3K-Akt and MAPK signaling pathways, both of which are involved in adipocyte differentiation and development [48]. For meat-quality traits, CP showed enrichment in lipid-related pathways, including fatty acid biosynthesis, elongation, and degradation [49], likely reflecting shared genetic influences on meat composition rather than a direct mechanistic effect of lipid metabolism on protein deposition. TF was enriched in the PI3K-Akt signaling pathway (*P* < 0.05), which has a critical role in adipogenesis [50], as well as in the VEGF signaling pathway, further supporting the link between vascular function and fat formation [51]. Fatty acid traits (e.g., C20:4, C18:2) were also enriched in key pathways known to regulate fatty acid metabolism, including Fatty acid biosynthesis (*P* < 0.01), Peroxisome, and the JAK-STAT signaling pathway [52, 53]. Overall, these pathway enrichments provide a biology-consistent context for interpreting the trait-associated gene signals identified in our analyses.

### Single-cell transcriptomic atlas of eight tissues in cattle

**Fig. 3:**
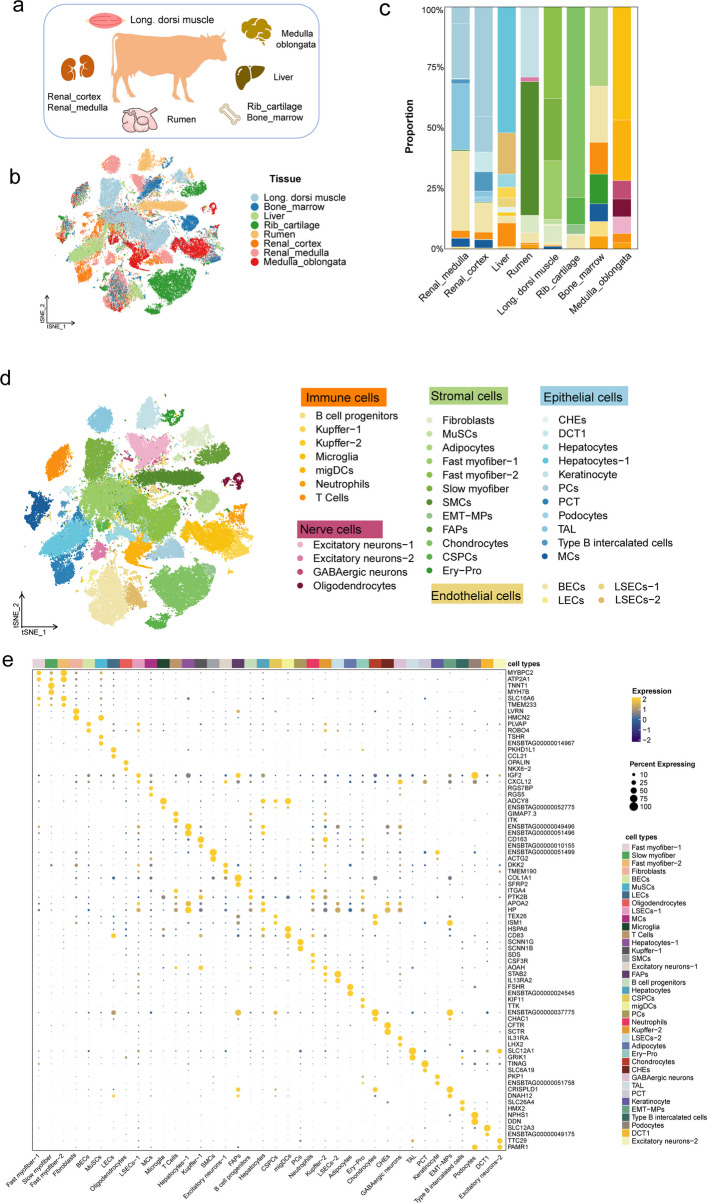
Single-cell transcriptomic profiling of eight tissues in cattle. **a** Schematic illustration of the eight tissues sampled for scRNA-seq, including longissimus dorsi muscle, medulla oblongata, renal cortex, renal medulla, liver, rib cartilage, bone marrow and rumen. **b** t-SNE visualization of all cells, colored by tissue of origin. **c** Bar plot showing the relative abundance of each cell type across tissues; colors are consistent with those used in d. **d** t-SNE visualization of all cells, colored by annotated cell type. In total, 38 cell types were identified and grouped into five major lineages: immune, epithelial, neuronal, endothelial and stromal cells. **e** Dot plot showing the expression level and proportion of cells expressing the top two marker genes for each cell type. Long. dorsi muscle = longissimus dorsi muscle

To characterize the cellular composition of tissues regulating key agronomic traits, we performed scRNA-seq or snRNA-seq on eight tissues from two adult Huaxi cattle using the 10× Genomics platform, including medulla oblongata, liver, rumen, longissimus dorsi muscle, bone marrow, rib cartilage, renal cortex and renal medulla (Fig. 3a and Table S6). In total, 98,314 cells/nuclei were initially captured, with an average sequencing depth of more than 360 million raw reads per tissue (Table S6). After stringent quality control (Fig. S2 and S3), 72,736 high-quality cells/nuclei were retained for downstream analyses. By integrating all high-quality cells across tissues using Seurat [34] (Fig. 3b and Fig. S4a, b), we annotated 38 distinct cell types based on canonical marker genes and grouped them into five major lineages: immune cells (*n* = 10,776), endothelial cells (*n* = 8,518), epithelial cells (*n* = 15,898), stromal cells (*n* = 32,176) and nerve cells (*n* = 5,368), with stromal cells comprising the largest fraction (44%) (Fig. 3d, Fig. S4c, and Table S8). The distribution of cell types revealed two general patterns. First, most cell types displayed high tissue specificity, consistent with their specialized functions. For example, hepatocytes specifically expressed *APOA2* and *ASGR1* [54], podocytes expressed *NPHS1* and *NPHS2* [55], and neuronal cells expressed *SYT1* (Fig. 3e, Fig. S5, and Table S9). Second, several common cell types, including immune cells, endothelial cells and fibroblasts, were broadly shared across multiple tissues (Fig. 3c). Cell-cycle analysis further revealed differences in functional states across lineages: immune cells showed a higher proportion of cells in S and G2/M phases, indicative of active proliferation, whereas epithelial, stromal and endothelial cells were predominantly in G0/G1 phase, suggesting an overall quiescent or low-proliferative state (Fig. S4d and Table S10).

### Single-cell-based prioritization of trait-associated cell types

**Fig. 4:**
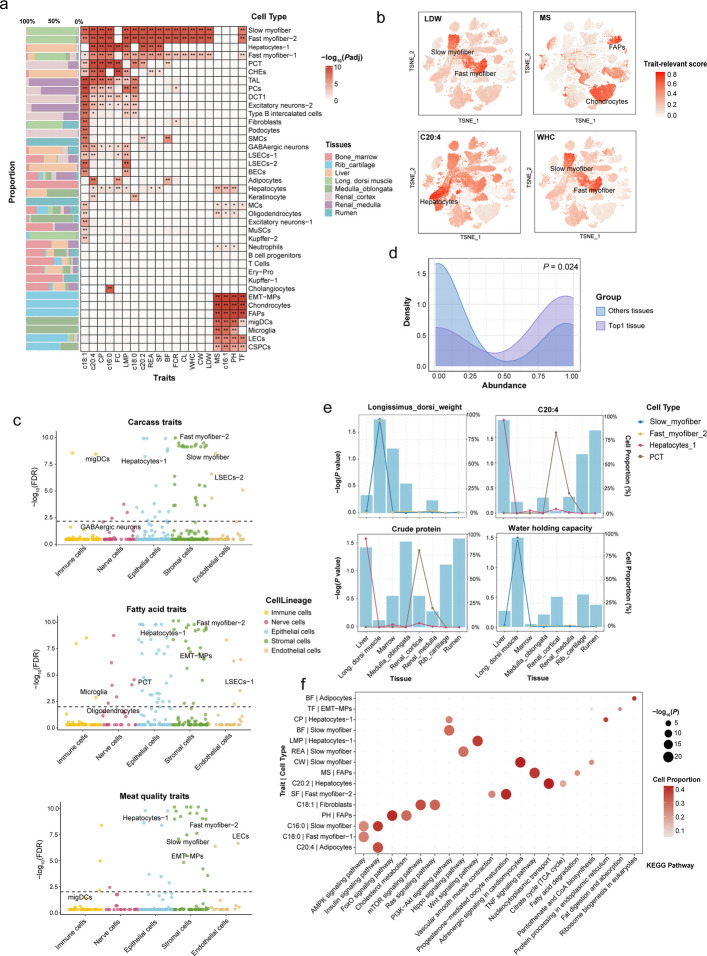
Enrichment analysis between cell types and complex traits. **a** Heat map showing associations between 38 cell types and 20 agronomic traits. The right panel shows the relative abundance of each cell type across tissues. Significance of trait–cell-type enrichment was estimated using scPagwas. Color intensity indicates enrichment strength (–log_10_(*P*_adj_), BH-FDR–adjusted *P* values). ^*^P_adj_ < 0.05, ^**^P_adj_ < 0.01. **b** t-SNE visualization of cell-level TRS for four representative traits (LDW, MS, C20:4 and WHC). Colors indicate annotated cell types, and cells with high TRS highlight trait-associated cell populations. **c** Enrichment of three major trait categories (carcass, fatty-acid and meat-quality traits; top to bottom) across five major cell lineages (immune, mesenchymal, epithelial, endothelial and neuronal). **d** Density plots showing the abundance distribution of the top two trait-associated cell types in the most trait-relevant tissue versus all other tissues. **e** Proportion of the top two trait-associated cell types across eight tissues. Bar height (left *y* axis) indicates trait–tissue enrichment (MAGMA), and points (right *y* axis) indicate the proportional abundance of the corresponding cell types. **f** scPagwas-based pathway-activity analysis for selected traits and their significantly associated cell types. Bubble size denotes the proportion of cells involved in each pathway and color represents pathway activity level. Long. dorsi muscle = longissimus dorsi muscle

To resolve the cellular contexts underlying economically important traits, we integrated GWAS results with single-cell transcriptomes from 38 annotated cell types using scPagwas [35]. The analyzed traits included carcass (*n* = 9), meat quality (*n* = 5), and fatty acid composition traits (*n* = 6). Preliminary analysis revealed that myofibers were associated with most carcass traits, whereas hepatocytes were strongly linked to several fatty-acid traits (e.g., C16:0 and C20:4) (Fig. 4a and Table S11).

The analysis first revealed significant associations between different categories of traits and specific cell types. For carcass traits, myofibers emerged as the most significantly associated cell type for the majority of traits (Fig. 4a). Specifically, slow myofibers showed extremely strong associations with LMP (*P* = 2.21 × 10^−11^), REA (*P* = 1.57 × 10^−11^), FCR (*P* = 2.76 × 10^−11^), CW (*P* = 2.95 × 10^−11^), and LDW (*P* = 2.97 × 10^−11^) (Fig. 4a). Notably, MS displayed a distinct association profile compared with other carcass traits, showing the strongest enrichment in EMT‑activated mesenchymal progenitor (EMT-MPs, *P* = 3.87 × 10^−12^), fibro/adipogenic progenitors (FAPs, *P* = 1.20 × 10^−11^), and lymphatic endothelial cells (LECs) (*P* = 5.76 × 10^−8^). FC was associated with hepatocytes (*P* = 9.78 × 10^−12^) and with epithelial cell types, including cholangiocytes (CHEs) (*P* = 1.94 × 10^−4^) and proximal tubular cells (PCT) (*P* = 5.43 × 10^−12^) (Fig. 4a). Among meat-quality and fatty-acid traits, TF, pH and C16:1 exhibited the strongest associations with EMT-MPs (*P* = 3.51 × 10^−12^) and were also significantly linked to FAPs (TF: *P* = 1.44 × 10^−11^; pH: *P* = 1.20 × 10^−11^; C16:1: *P* = 1.41 × 10^−11^) (Fig. 4a). Most fatty acid traits, with the exception of C16:1, showed significant enrichment in myofibers, suggesting that myofiber-related genetic programs may contribute to variation in intramuscular fatty acid composition [56, 57]. In addition to this myofiber-related pattern, key fatty acid traits C16:0 (*P* = 9.58 × 10^−12^), C20:4 (*P* = 8.26 × 10^−12^), and C20:2 (*P* = 5.04 × 10^−11^), together with CP (*P* = 8.88 × 10^−12^), were significantly associated with hepatocytes, reflecting the central role of the liver in lipid and protein metabolism [53, 58]. Meat quality indicators such as water-holding capacity (WHC) and shear force (SF) were also significantly associated with myofibers, underscoring the crucial contribution of muscle cells to meat-quality formation [59]. The cell-trait relevance score (TRS) analysis further confirmed that distinct cell types were specifically associated with individual traits: myofibers showed high TRS values for LDW and WHC, FAPs and chondrocytes for marbling score, and hepatocytes for C20:4 (Fig. 4b and Fig. S6). Finally, enrichment analysis at the level of the five major cell lineages and three main trait categories revealed that carcass, meat-quality and fatty-acid traits were predominantly associated with mesenchymal and epithelial lineages, with selected immune cell types (migDCs and microglia) also contributing to trait formation (Fig. 4c).

To investigate the tissue localization of trait-associated cell types, we next performed cell abundance analysis. Compared to the most trait-relevant tissues, other tissues showed lower abundance of the top two trait-associated cell types, indicating that these cells are typically enriched in their corresponding trait-relevant tissues (Wilcoxon rank-sum test, *P* = 0.024) (Fig. 4d). For LDW and REA, the key cell type—myofibers—displayed highest abundance in the longissimus dorsi muscle, the core trait-relevant tissue (Fig. 4e), supporting its central role in trait formation. Similarly, pH-associated cells were highly enriched in kidney and rib cartilage tissues (Fig. S7), in agreement with our tissue-level observations (Fig. 2c), suggesting that cartilage tissue contributes substantially to this trait. However, we also observed evidence of cross-tissue regulation. For CP and C20:4, their major associated cell types (hepatocytes and PCT) did not show the highest abundance across all trait-relevant tissues (Fig. 4e), suggesting that cellular regulation of traits is not necessarily restricted to a single tissue but may rely on coordinated networks spanning multiple organs [13, 60].

Pathway-activity analysis using scPagwas further revealed potential signaling routes through which cell types influence trait formation. Myofibers may regulate backfat thickness (BF), CW, and REA through the PI3K-Akt signaling pathway (*P* < 0.01), pantothenate and CoA biosynthesis (*P* = 1.38 × 10^−4^), and the Hippo signaling pathway (*P* < 0.01), respectively. Trait formation, however, often involves the coordinated action of multiple cell types. For instance, BF was significantly associated with adipocytes (*P* = 6.43 × 10^−6^), and studies in pigs have shown that backfat thickness correlates with adipocyte volume and lipid-metabolism gene modules [61, 62]. LMP was likewise influenced by hepatocytes (*P* = 1.33 × 10^−10^), and was linked to distinct pathways such as ribosome biogenesis in eukaryotes (*P* = 0.00245) and the Wnt signaling pathway (*P* < 0.01) (Fig. 4f). Furthermore, myofibers regulated the metabolism of fatty acids including C16:0, C18:0, and C18:1 through signaling pathways such as AMPK (*P* = 1.00 × 10^−19^), insulin (*P* = 5.16 × 10^−19^), and mTOR [63] (*P* = 1.00 × 10^−20^). Fast-twitch myofibers may additionally influence meat tenderness via pathways related to vascular smooth muscle contraction (*P* = 1.90 × 10^−9^) and progesterone-mediated oocyte maturation (*P* = 1.00 × 10^−20^) (Fig. 4f).

To assess whether the enrichment results were influenced by cell count bias, we performed a robustness analysis based on subsampling 200 cells per cell type (Fig. S8a). The resulting enrichment scores were highly consistent with those obtained from the full dataset (Pearson’s *r* = 0.75, *P* = 2.20 × 10^−16^) (Fig. S8b). Trait-level subsampling further showed that most traits exhibited high concordance (*r* > 0.75), with correlation coefficients ranging from 0.35 for REA (*P* = 0.034) to 1.0 for MS (*P* < 2.20 × 10^−16^), confirming the robustness of our cell–trait associations (Fig. S8c).

Identifying key cell types underlying complex traits is essential, as it provides the cellular context needed to interpret the genetic regulatory architecture of important cattle traits and paves the way for more precise, cell-targeted breeding interventions.

### DEG profiles and regulatory networks of TFs in myofibers reveal their central role

**Fig. 5:**
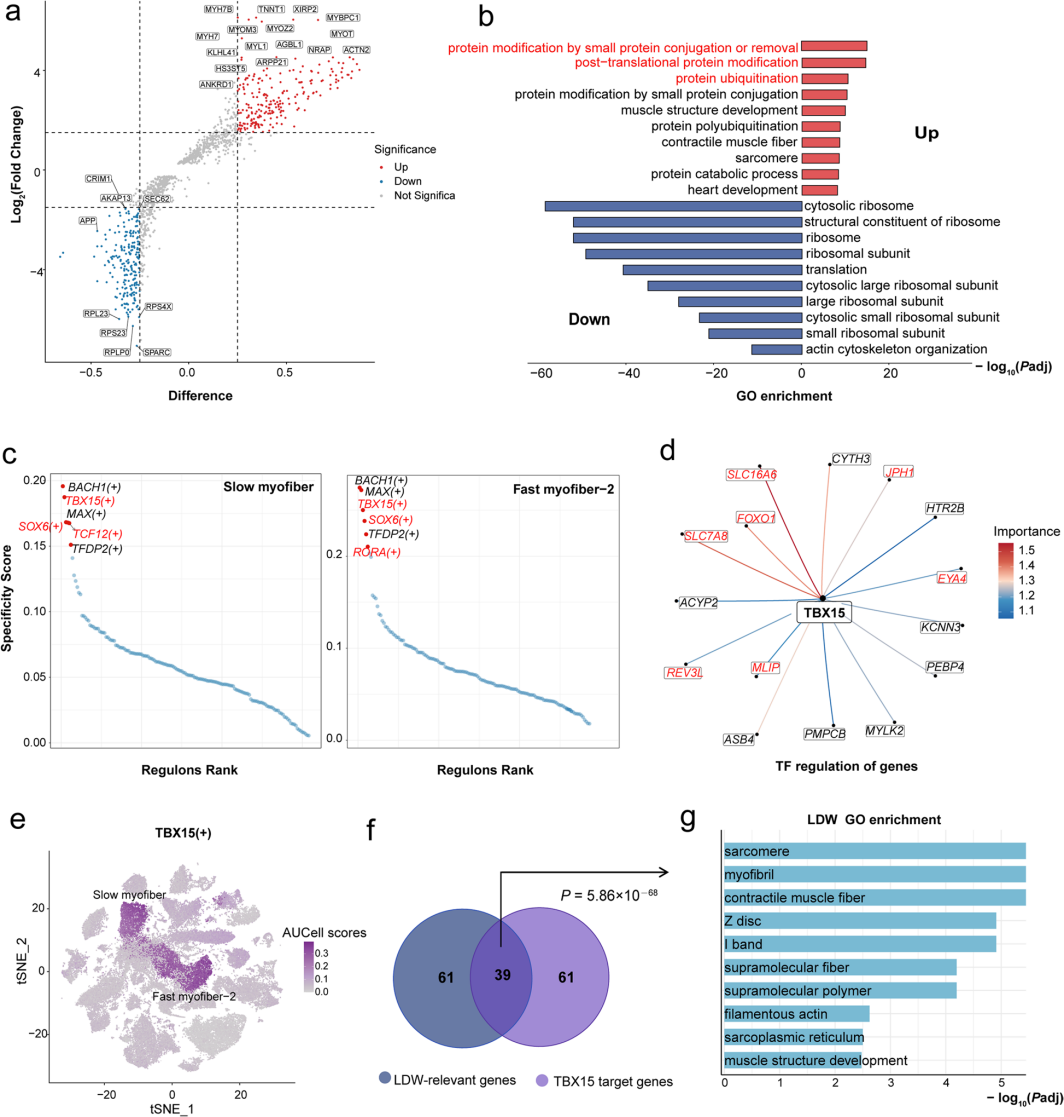
Transcriptional regulation and functional genomics of myofibers in cattle. **a** Volcano plot showing differentially expressed genes in myofibers compared with all other cell types. Red and blue points indicate significantly upregulated and downregulated genes, respectively. **b** GO enrichment analysis of upregulated (red) and downregulated (blue) genes from a. Terms are grouped into processes related to protein modification and muscle structure development. **c** TFs specificity scores in slow (left) and fast (right) myofibers. Muscle-related TFs are highlighted in red. **d** TBX15-centred gene regulatory network. The top 15 target genes ranked by importance score are shown; genes overlapping with LDW–associated genes are highlighted in red. **e** AUCell scores for TBX15 regulon activity across all cells. **f** overlap between LDW-associated genes (identified by scPagwas) and TBX15 target genes. P value is from a hypergeometric test. **g** GO enrichment analysis of genes overlapping between LDW-associated genes and TBX15 targets, showing enrichment for processes related to muscle formation, organization, maintenance and remodeling

Given the strong associations between myofibers and multiple traits, we first investigated their central role in skeletal muscle biology through differential expression analysis. Compared with other cell types, myofibers showed marked upregulation of skeletal muscle–characteristic genes, including *MYH7B*, *MYH7* and *MYBPC1* (Fig. 5a and Table S12). Functional enrichment analysis indicated that these differentially expressed genes were mainly involved in key processes such as protein modification (e.g., modification by small protein conjugation or removal) and muscle structure development (Fig. 5b), these enriched terms were consistent with myofiber-specific functional features.

We next performed transcription factors (TFs) analysis to dissect the genetic regulatory programs of myofibers. In slow myofibers, we identified TBX15, SOX6 and TCF12 as key TFs, whereas TBX15, SOX6 and RORA were specifically enriched in fast myofibers (Fig. 5c). TCF12 activity was also detected in fibroblasts (Fig. S9a). Notably, TBX15 and SOX6 exhibited high activity in both slow and fast myofibers (Fig. 5e and Fig. S9b), suggesting that they may serve as core regulators of myofiber identity. Consistent with this interpretation, previous studies have implicated TBX15 and SOX6 in muscle fiber-type specification [64, 65] and TCF12 in MyoD-mediated myogenic gene regulation [66].

Regulatory network analysis further indicated that TBX15 may mediate skeletal muscle growth by regulating genes such as *SLC16A6* and *SLC7A8* (Fig. 5d and Table S13). To explore the regulatory mechanisms underlying LDW, we performed functional enrichment on the overlapping set of genes between trait-associated genes (Table S14) and TBX15 target genes. These common genes were predominantly involved in the formation, organization, maintenance and remodeling of muscle at both structural and functional levels, including myofibril assembly, muscle structure development and contractile muscle fiber terms (Fig. 5f). Together, these findings systematically highlight the central role of skeletal muscle cells in cattle growth and delineate their multilayered regulatory basis.

### Hepatic and microglial regulation of fatty-acid traits

**Fig. 6:**
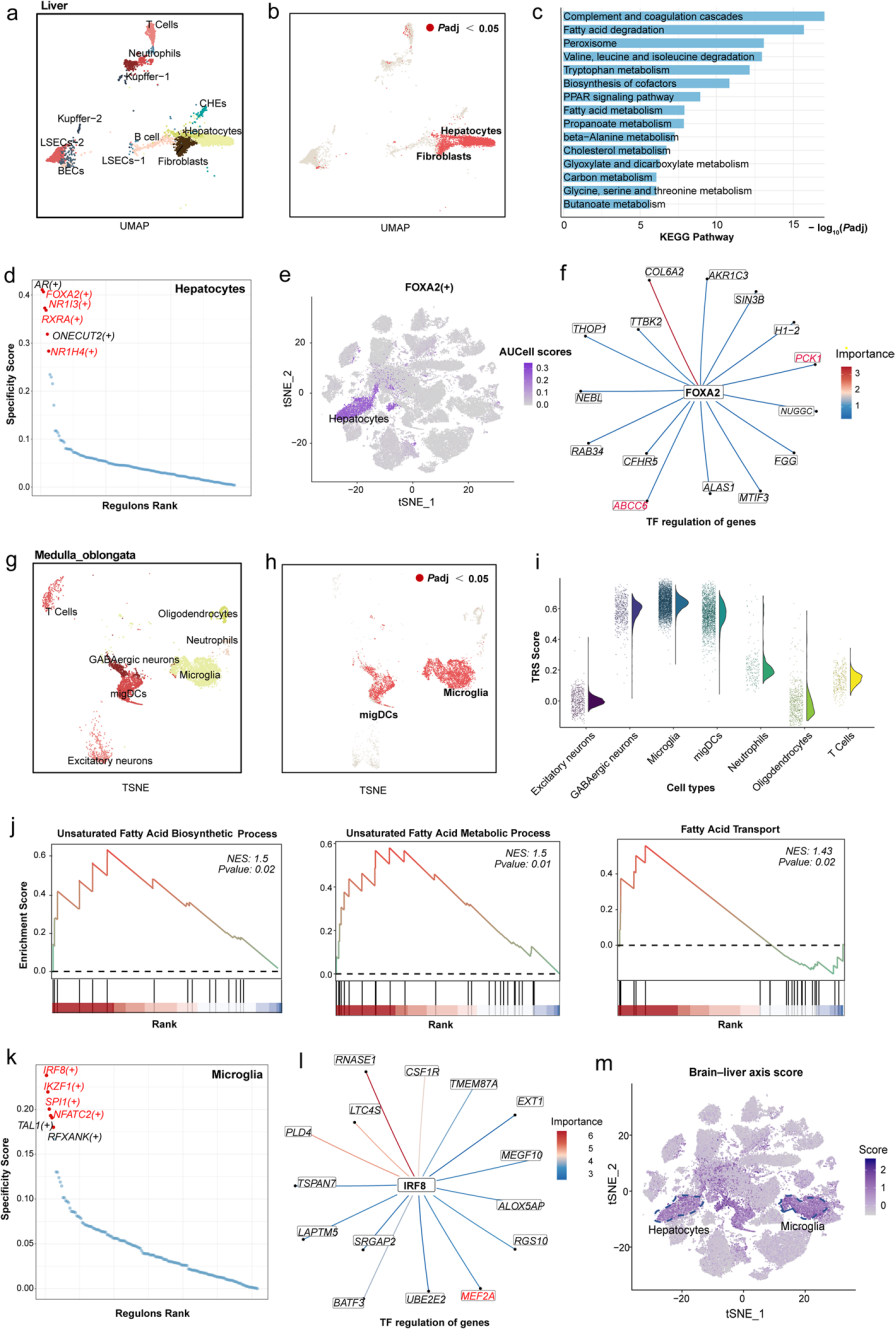
Single-cell integration of GWAS signals for fatty acid traits C16:0 and C16:1. **a** UMAP of liver cells annotated into nine major cell types. **b** scPagwas cell-type enrichment for C16:0 by integrating C16:0 GWAS summary statistics with liver single-cell data; significant associations were defined as *P*_adj_ < 0.05 (two-sided hypergeometric test with FDR correction). **c** KEGG enrichment of upregulated DEGs in hepatocytes; *x*-axis indicates −log(adjusted *P* value). **d** Hepatocyte transcription-factor specificity scores, with hepatocyte-associated TFs highlighted in red. **e** AUCell scores of FOXA2 regulon activity across cell types. **f** FOXA2-centered regulatory network showing the top 15 target genes; red genes overlap with C16:0-associated genes. **g** t-SNE of medulla oblongata cells annotated into seven major cell types. **h** scPagwas enrichment for C16:1 using medulla oblongata single-cell data; significant associations were defined as in **b**. **i** Distribution of trait relevance scores for C16:1 across cells. **j** GO GSEA of microglial DEGs ranked by log₂(fold change), using clusterProfiler and 10,000 one-sided permutations. **k** Microglial TF specificity scores, with microglia-associated TFs highlighted in red. **l** IRF8-centered regulatory network showing the top 15 targets; *MEF2A* marks overlap with C16:1-associated genes. **m** Brain–liver axis gene scores; labeled cell types show relatively high scores

Given the liver’s critical role in regulating multiple fatty acid traits, scPagwas-based enrichment analysis identified a significant association between hepatocytes and C16:0 (*P* = 9.58 × 10^−12^; Fig. 4a), prioritizing hepatocytes as a key cell type implicated in the genetic architecture of fatty-acid traits. Focusing on liver tissue, we identified nine major cell populations, including hepatocytes, myofibers, and cholangiocytes (CHEs) (Fig. 6a). Consistent with multiple tissue-level scRNA-seq data, hepatocytes exhibited the strongest association with C16:0 (*P* = 4.39 × 10^−9^; Fig. 6b). Differential expression analysis of hepatocytes showed that upregulated genes were predominantly enriched for fatty acid degradation, fatty acid metabolism, peroxisome, PPAR signaling, and cholesterol metabolism pathways (Fig. 6c), consistent with hepatocytes’ established involvement in lipid metabolism [67].

Transcription factor analysis further identified FOXA2, NR1H4, RXRA and NR1I3 as key transcriptional regulators closely linked to hepatocyte identity and metabolic function (Fig. 6d). These factors showed high activity specifically in hepatocytes (Fig. 6e and Fig. S9c). By intersecting FOXA2-regulated genes (Table S15) with C16:0-associated genes (Table S16), we identified several common genes, including *ABCC6*,* PCK1*, and *DPYS* (Fig. 6f and Fig. S9e). These genes are involved in hepatic ATP/PPi homeostasis, gluconeogenesis and glucose–lipid balance, and pyrimidine degradation, respectively [68–70]. Additionally, the regulons of other transcription factors associated with C16:0 traits intersected with the C16:0-associated gene set at three key genes—*CES3*, *CUX2* and *PPARA* (Fig. S9d and f), which are involved in lipid hydrolysis, energy metabolism and fatty acid oxidation, respectively. These findings suggest that C16:0-related genetic effects are likely mediated through multiple hepatic metabolic pathways.

Emerging studies highlight a bidirectional liver–brain axis in which autonomic signaling and hepatokines modulate hepatic lipid metabolism and energy homeostasis [71, 72]. For example, the liver-derived hormone *FGF21* enhances hepatic sympathetic nerve activity via the brain–liver axis, thereby remodeling hepatic lipid metabolism and reversing fatty liver disease [73]. Motivated by these findings, we investigated whether specific central nervous system cell types contribute to fatty-acid trait regulation, particularly the fatty acid C16:1. Cell-type enrichment analysis identified microglia as a candidate regulatory cell type (Fig. 4a). To further examine this result, we placed emphasis on the scRNA-seq data of medulla tissue and identified seven major cell types, including neurons, microglia, and T cells (Fig. 6g). scPagwas analysis further supported a microglial association with C16:1 (*P* = 3.56 × 10^−7^; Fig. 6h and i), consistent with a potential involvement of microglia in C16:1-related processes. GSEA of differentially expressed genes in microglia revealed significant enrichment in pathways related to unsaturated fatty acid metabolism, biosynthesis, and fatty acid transport (Fig. 6j), consistent with lipid-metabolic activity in microglia that may be relevant to C16:1. Transcription factor analysis identified key regulators of microglial identity, including IRF8, IKZF1, SPI1, and NFATC2 [57–60] (Fig. 6k), which show highly specific activity in microglia (Fig. S9g). Further integrative analysis revealed *MEF2A* as a common gene between microglia-associated genes and C16:1-associated genes (Fig. 6g and Table S17, S18). Given the reported involvement of MEF2 family members in immune–metabolic regulation, including fatty acid and triglyceride synthesis [74], these findings suggest that MEF2A may link microglial regulatory programs to C16:1-associated genetic signals.

To further explore potential liver–brain coordination, we curated a candidate liver–brain axis gene set—including *APOE* and *TTR*—that has reported roles in liver and CNS metabolic or inflammatory pathways [71, 75–78]. Module scoring revealed preferential enrichment of this signature in hepatocytes and microglia (Fig. 6m), consistent with the possibility that hepatocytes and microglia represent peripheral and central components, respectively, of a putative liver–brain axis [79].

Collectively, these results suggest distinct but complementary roles of hepatocytes and microglia in the genetic regulation of fatty acid traits, with hepatocytes more closely associated with C16:0 regulation and microglia with C16:1 regulation. These findings support a potential liver–brain regulatory framework linking peripheral lipid metabolism with central immune-related responses.

## Discussion

Translating trait-associated loci identified by GWAS into specific genes, relevant cell types, and ultimately mechanistic insight remains a major challenge [22, 80]. To address this gap, integrative strategies that incorporate molecular phenotype data—such as tissue- or cell type-resolved gene expression—have been widely adopted. By bridging the genotype-to-molecular-phenotype layer, these approaches enhance the prioritization of candidate genes and clarify the biological contexts in which genetic variants exert their effects [27]. Among the existing frameworks, gene-based association tests and the integration of GWAS with molQTL datasets are two of the most commonly applied strategies. Notably, molQTL-based approaches not only improve the identification of putative causal genes but also offer insight into the regulatory mechanisms underlying genetic associations. Moreover, sc-eQTL analyses have extended these efforts by resolving cell-type-specific regulatory variants in recent years [81]. The most influential efforts include the GTEx project in humans and its livestock counterpart, FarmGTEx, both of which provide essential resources for interpreting GWAS signals within appropriate molecular, tissue, and cellular contexts [5, 6, 9].

A major limitation of molQTL-based approaches is the need for large, genotype-matched molecular phenotype datasets across multiple tissues and biological contexts. The GTEx project (v8) exemplifies this scale, comprising 838 donors and 17,382 RNA-seq samples spanning 54 tissue sites [5]. Comparable efforts in livestock species, the PigGTEx includes 5,457 samples from 34 tissues; ChickenGTEx aggregates 7,015 RNA-seq samples across 28 tissues; and SheepGTEx analyzed 6,761 samples from 51 primary tissues [7, 8, 82]. In cattle, the CattleGTEx integrates 7,180 RNA-seq samples and establishes genetic regulatory maps across 23 tissues—most of which are enriched for reproductive and lactation traits (e.g., mammary gland, uterus, ovary) [9]. For single-cell datasets, high experimental cost, technical variation, incomplete annotation, and limited sample sizes further restrict the broad implementation of sc-eQTL in livestock species.

Given the incomplete definition of trait-relevant tissues for meat production in beef cattle and the limited availability of corresponding molecular phenotype data, we employed a gene-based association framework to integrate population-level GWAS signals with bulk and single-cell RNA-seq data. This strategy allowed us to infer trait-relevant tissues and cell types, identify key transcriptional regulators and their downstream targets, and delineate biological processes contributing to the formation of complex traits in beef cattle.

### Tissue-level regulatory insights

We first identified tissues most strongly associated with beef cattle production traits by integrating GWAS signals and transcriptome data. Beyond confirming well-established observations, such as the enrichment of C16:0 and C20:4 fatty acid trait signals in the liver, consistent with its central role in lipid synthesis [58]. We uncovered a novel association: significant enrichment of FCR signals within the liver. This finding aligns with functional studies showing that the liver-specific protein FABP1 promotes triglyceride synthesis and VLDL secretion in bovine hepatocytes, thereby facilitating fat deposition [83], suggesting a potentially important hepatic pathway underlying FCR. We also observed that multiple traits were enriched in nervous system–related tissues, including the brain, cerebellum, and medulla oblongata. Although previous research has indicated roles for these tissues in regulating body composition, metabolism, and behavior [84, 85], systematic evidence directly linking neural tissues to specific agronomic traits in beef cattle has been limited. Here, we show that CL is associated with the medulla oblongata and brain regions, and WHC exhibits enrichment in the brain, medulla oblongata, and hypothalamus (Fig. 2c). These observations highlight a potentially underappreciated role of the nervous system in shaping growth and production traits. Consistent with this nervous-system-related signal, *HTR3B*, which encodes a subunit of the serotonin-gated 5-HT3 receptor [86], emerged as an overlapping candidate for WHC, suggesting a potential link between GWAS signals for meat-quality traits and neuromodulatory cellular contexts.

### Cell-level regulatory insights

Building on these tissue-level findings, we generated single-cell transcriptome profiles from selected trait-relevant tissues. As expected, muscle fibers emerged as critical regulators of growth and carcass traits (Fig. 4a). Previous work in pigs has shown that a higher proportion of type IIx fibers in the longissimus lumborum is associated with increased REA and may contribute to rapid muscle growth and meat-quality variation [87, 88]. Consistent with these findings, myofibers in our dataset showed strong associations with multiple traits, underscoring their central role in trait determination [89].

Hepatocytes, the principal functional units of the liver, were also strongly associated with traits such as C20:4, C16:0, FC, and CP, consistent with their key roles in lipid and protein metabolism [58, 90]. In line with this hepatocyte-centered pattern, several recurrent C16:0-associated candidates also pointed to lipid handling and cellular lipid homeostasis, including *PLA2G12B*, *OSBPL2*, and *ATG2B*. In particular, *PLA2G12B* has been linked to endoplasmic reticulum lipid channeling and triglyceride-rich lipoprotein expansion [40], providing a plausible connection between hepatocyte-related regulatory signals and saturated fatty acid phenotypes. Intriguingly, kidney epithelial populations—including PCT and ascending LECs—displayed regulatory profiles similar to hepatocytes. This likely reflects shared transcriptional programs between liver and kidney, as supported by prior evidence that HNF1A and HNF4A regulate gene expression in both tissues [91, 92], in agreement with our transcription factor analyses (Fig. S9h). Moreover, traits such as MS, C16:1, pH, and TF exhibited shared regulatory patterns at the cellular level, possibly due to overlapping pathways in lipid deposition and muscle development. Prior studies have documented shared regulatory networks linking lipid metabolism with muscle growth and protein content [93–95], consistent with our co-enrichment observations. Nonetheless, functional experiments will be needed to validate these mechanistic hypotheses.

At cellular resolution, a notable discovery in our dataset was the potential role of microglia in regulating fatty acid traits. Palmitoleic acid-associated GWAS signals were enriched in microglia (Fig. 4a), consistent with the notion that microglia are responsive to lipid composition and can modulate their metabolic and inflammatory states in response to fatty acid cues [96, 97]. Our data further revealed that hepatocyte-upregulated genes were enriched for pathways related to fatty acid degradation, PPAR signaling, and peroxisome activity (Fig. 6c), core elements of hepatic β-oxidation and lipid handling that are central to metabolic liver disease progression [98, 99]. These findings suggest that microglial signals may represent neuroimmune responses to variation in fatty-acid metabolism, potentially mediated by indirect liver–brain communication pathways, rather than indicating a confirmed direct mechanism. This cellular pattern can be placed within the broader framework of the emerging liver–brain axis, in which bidirectional communication between the liver and the central nervous system—mediated by autonomic innervation, hepatokines, and lipid-derived metabolites—coordinates hepatic lipid accumulation and neuroinflammation [71, 73, 100]. We propose a conceptual model in which hepatocyte-derived circulating mediators (e.g., *FGF21*, APOE-related lipid/lipoprotein signals, and bile acids), together with autonomic/neuroendocrine pathways, may indirectly modulate microglial lipid handling and inflammatory tone [73]. In this context, while preliminary and correlative, the strong association of hepatocytes with saturated fatty-acid traits such as C16:0, together with the microglial association with the monounsaturated fatty acid C16:1, and the elevated liver–brain axis gene scores in both hepatocytes and microglia (Fig. 6m), suggest that distinct but related fatty-acid traits may be differentially handled by peripheral hepatic and central neuroimmune compartments. Given the central role of dysregulated liver–brain and brain–gut–liver axes in human metabolic liver disease, including MASLD and hepatic encephalopathy [79, 98], our findings in cattle offer valuable comparative insights and potential candidate pathways for human studies.

Taken together, the consistent cell-type association patterns across myofibers, hepatocytes, renal epithelial cells and microglia suggest that complex agronomic traits in cattle may reflect multi-tissue contributions rather than a single tissue acting in isolation. Although our analyses are correlative, they provide a cell-type–resolved starting point for future work to test whether hepatocyte-derived lipid signals and microglial immunometabolism are causally involved in fatty-acid trait variation.

### Regulatory influence beyond cell abundance

Notably, several trait-associated cell types were not the most abundant populations within their corresponding tissues (Fig. S7). For example, CW showed a top-ranked tissue enrichment signal in renal cortex in our bulk analysis, consistent with kidney’s key role in muscle anabolism through influencing intake of 1,25(OH)_2_-vitamin D [44], whereas the most CW-associated cell type in the single-cell analysis—myofibers—was predominantly found in skeletal muscle (Fig. S7). Similar discrepancies were observed for CP and C20:4, where hepatocytes and PCT cells were trait-relevant even though kidney exhibited only modest tissue-level association (Fig. 4e). These patterns emphasize that regulatory influence is not determined solely by cell abundance. Rare but highly specialized cell populations can disproportionately shape trait outcomes, as highlighted by integrative GWAS–single-cell analyses and studies leveraging scRNA-seq to identify rare but functionally critical cell states [21, 101, 102], and cross-tissue hierarchical regulation—where initiating-tissue variation affects effector tissues via metabolites or endocrine signaling—may also contribute [17]. Technical and analytical factors, including differences in data generation, cell-type composition estimates and statistical modeling, may further influence these patterns [103, 104]. Together, these observations highlight the need for a multidimensional framework that integrates tissue context, cell identity, abundance, and functional state.

### Transcription factor networks and functional mechanisms

Finally, transcription factor and functional enrichment analyses provided mechanistic insights into how trait-associated cell types influence phenotypes. Consistent with previous studies, we identified myofiber-specific regulators TBX15, SOX6, TCF12, and RORA [66, 105–107] (Fig. 5). We also identified CHE-specific regulators such as HNF1B, NR1H4, and HNF4A [108–110] (Figs. S9i and S10). HNF1B was additionally identified as a specific regulator in PCT cells and PCs (Fig. S10 and Table S19), consistent with its known roles in renal development, nephron segmentation, and ciliary function [111]. These findings reinforce the shared regulatory architecture between liver and kidney, mirroring their similar patterns of trait association. Moreover, scPagwas enrichment of TFs target genes identified *ATG2B*, *OSBPL2*, and *PLA2G12B*, genes implicated in lipid and fatty acid metabolism as candidates underlying palmitic acid–related traits [40, 112, 113]. This suggests that these genes represent plausible regulators of fatty acid metabolic pathways and may serve as promising targets for future functional studies in livestock models.

### Limitations and future directions

Our integration of GWAS with transcriptomic data reveals potential regulatory mechanisms underlying complex traits in beef cattle at both tissue and cellular levels, but several limitations and future directions should be pronounced. First, tissue- or cell-type associations derived from statistical enrichment do not establish causality and may reflect indirect relationships, as noted in previous frameworks [15, 20, 30, 35, 102], thus, the observed liver–brain coordination in this study should be interpreted as correlative rather than mechanistic evidence. As high-throughput technologies expand molecular phenotype datasets for meat production–relevant tissues, integrating GWAS with more comprehensive molQTL resources will be complementary for prioritizing functional genes and elucidating the molecular mechanisms underlying trait variation. Second, SNP-to-gene mapping using a fixed ± 50 kb window is a pragmatic approximation and may overlook distal regulatory targets of noncoding GWAS variants; future studies could refine variant-to-gene assignments by incorporating molQTL and functional genomics data, such as chromatin interaction maps [114, 115]. Third, for some bulk tissues, limited biological replication may increase uncertainty in tissue-specific expression estimates and enrichment results. Similarly, our single-cell transcriptomic profiling was conducted on only two cattle across eight tissues; although the total cell numbers are substantial, this limited biological replication (*n* = 2) may reduce the robustness of cell-type abundance estimates and limit the generalizability of downstream inferences. To mitigate these limitations, we emphasize cross-method concordance, apply BH-FDR correction, and interpret marginal signals with caution. Fourth, current datasets largely represent adult tissues and therefore miss key developmental cell states. Genetic effects acting during early development may have a larger influence on adult phenotypes, underscoring the value of developmental GTEx-like frameworks that incorporate multi-tissue molecular phenotypes across developmental stages [116]. Fifth, existing approaches do not fully integrate multi-layered omics, including scATAC-seq [117], spatial transcriptomics [118], and epigenetic data, which are essential for linking regulatory elements to target genes and trait-relevant cell types. Future studies should leverage functional genomics, spatially resolved single-cell technologies, and cross-species comparisons [119] to build more complete regulatory maps. Finally, experimental validation remains essential. CRISPR-based perturbation (e.g., CRISPR interference) [120] and cellular models of lipid metabolism and muscle development will help establish causal links between genetic variants, cellular functions, and phenotypic outcomes, providing a stronger mechanistic basis for livestock genetic improvement.

## Conclusion

In this study, we delineated the regulatory architecture of 20 meat production traits in beef cattle at the tissue and cell levels and linked specific tissues and cell types to phenotypic variation. We identified key trait-associated cells, their core transcriptional regulators, downstream pathways and coordinated cross-tissue mechanisms, providing insight into the mechanisms by which genetic variation influences economically important traits. These cell-resolved findings offer a valuable resource for future mechanistic studies, inform the design of cell-informed precision breeding strategies, and guide the selection of tissues and cell types for subsequent large-scale molecular phenotyping.

## Supplementary Information

Additional file 1: Table S1. GWAS summary statistics for the 20 agronomic traits. Table S2. Genome-wide significant SNPs identified for the 20 agronomic traits. Table S3. Annotation of trait-associated SNPs and nearby genes within ± 50 kb. Table S4. Sample information and classification of tissue sample. Table S5. Top 10% tissue-specific genes. Table S6. Overview of cattle tissue sampling and sc/snRNA-seq data quality. Table S7. Genomic inflation factors (λGC) for the GWAS summary statistics. Table S8. Summary of cell types and cell numbers across major cell categories. Table S9. Marker genes for each cell type. Table S10. Cell cycle phase distribution across cell types. Table S11. Full TRS results, including raw *P* values and −log10(*P*_adj_) values. Table S12. Differentially expressed genes between fast and slow myofibers. Table S13. Target genes of myofiber-associated transcription factors. Table S14. LDW-associated genes and Pearson correlation coefficients. Table S15. Target genes of hepatocyte transcription factors. Table S16. C16:0-associated genes and Pearson correlation coefficients. Table S17. C16:1-associated genes and Pearson correlation coefficients. Table S18. Target genes of microglia-associated transcription factors. Table S19. Top 10 transcription factors per cell type

Additional file 2: Fig. S1. Quantile–quantile plots for agronomic traits. Fig. S2. Barcode Rank Plot for Cell Calling. Fig. S3. Quality-control metrics across eight tissues. Fig. S4. Integration of single-cell atlases and cell-type composition across eight tissues. Fig. S5. Marker gene expression for selected cell types. Fig. S6. Cell-level trait relevance scores for agronomic traits. Fig. S7. Tissue distribution of top trait-associated cell types. Fig. S8. Robustness of cell–trait associations under random subsampling. Fig. S9. Transcription factor regulatory activity across cell types. Fig. S10. Transcription factor specificity across cell types

## Data Availability

The GWAS summary statistics generated in this study have been deposited in Figshare and are publicly available through Figshare (10.6084/m9.figshare.32567283). Public GWAS summary statistics for 10 agronomic traits in Chinese Simmental cattle were obtained from a previous study and are available via Figshare (https://doi.org/10.6084/m9.figshare.20032988.v1). Individual-level data (including genotype/phenotype data and raw single-cell sequencing data) are not publicly available due to data-owner/usage restrictions. Access to these controlled datasets may be granted upon reasonable request to the corresponding author and, where applicable, subject to approval by the data owner and completion of required data use agreements.

## Abbreviations

BF: Backfat thickness
CHEs: Cholangiocytes
CP: Crude protein
CW: Carcass weight
DESE: Driver-tissue estimation by selective expression
EMT-MPs: EMT-activated mesenchymal progenitors
FAPs: Fibro/adipogenic progenitors
Fast myofiber: Fast-twitch muscle fiber
FC: Fat color
FCR: Fat coverage rate
GSEA: Gene set enrichment analysis
GWAS: Genome-wide association study
LD: Linkage disequilibrium
LDW: Longissimus dorsi weight
LECs: Lymphatic endothelial cells
LMP: Lean meat percentage
MAD: Median absolute deviation
MAGMA: Multi-marker analysis of genomic annotation
MASLD: Metabolic dysfunction-associated steatotic liver disease
migDCs: Migratory dendritic cells
MS: Marbling score
PCA: Principal component analysis
PCC: Pearson correlation coefficient
PCs: Principal cells
PCT: Proximal tubular cells
REA: Rib-eye area
scATAC-seq: Single-cell assay for transposase-accessible chromatin using sequencing
scPagwas: Single-cell pathway-based polygenic regression for GWAS
scRNA-seq: Single-cell RNA sequencing
SF: Shear force
Slow myofiber: Slow-twitch muscle fiber
snRNA-seq: Single-nucleus RNA sequencing
TF: Total fat content
TFs: Transcription factors
TRS: Trait relevance score
WHC: Water-holding capacity
