# Supplementary material for: Integrating bulk and single-cell RNA sequencing with GWAS reveals regulatory networks underpinning complex traits in beef cattle
Source: J Anim Sci Biotechnol. 2026 Jul 28;17:151. doi: 10.1186/s40104-026-01471-2 (PMC13411826; doi:10.1186/s40104-026-01471-2)
Supplement: Supplementary file 2 — Additional file 2: Fig. S1. Quantile–quantile plots for agronomic traits. Fig. S2. Barcode Rank Plot for Cell Calling. Fig. S3. Quality-control metrics across eight tissues. Fig. S4. Integration of single-cell atlases and cell-type composition across eight tissues. Fig. S5. Marker gene expression for selected cell types. Fig. S6. Cell-level trait relevance scores for agronomic traits. Fig. S7. Tissue distribution of top trait-associated cell types. Fig. S8. Robustness of cell–trait associations under random subsampling. Fig. S9. Transcription factor regulatory activity across cell types. Fig. S10. Transcription factor specificity across cell types [file 40104_2026_1471_MOESM2_ESM.docx]

# **Supplementary Figures**

| 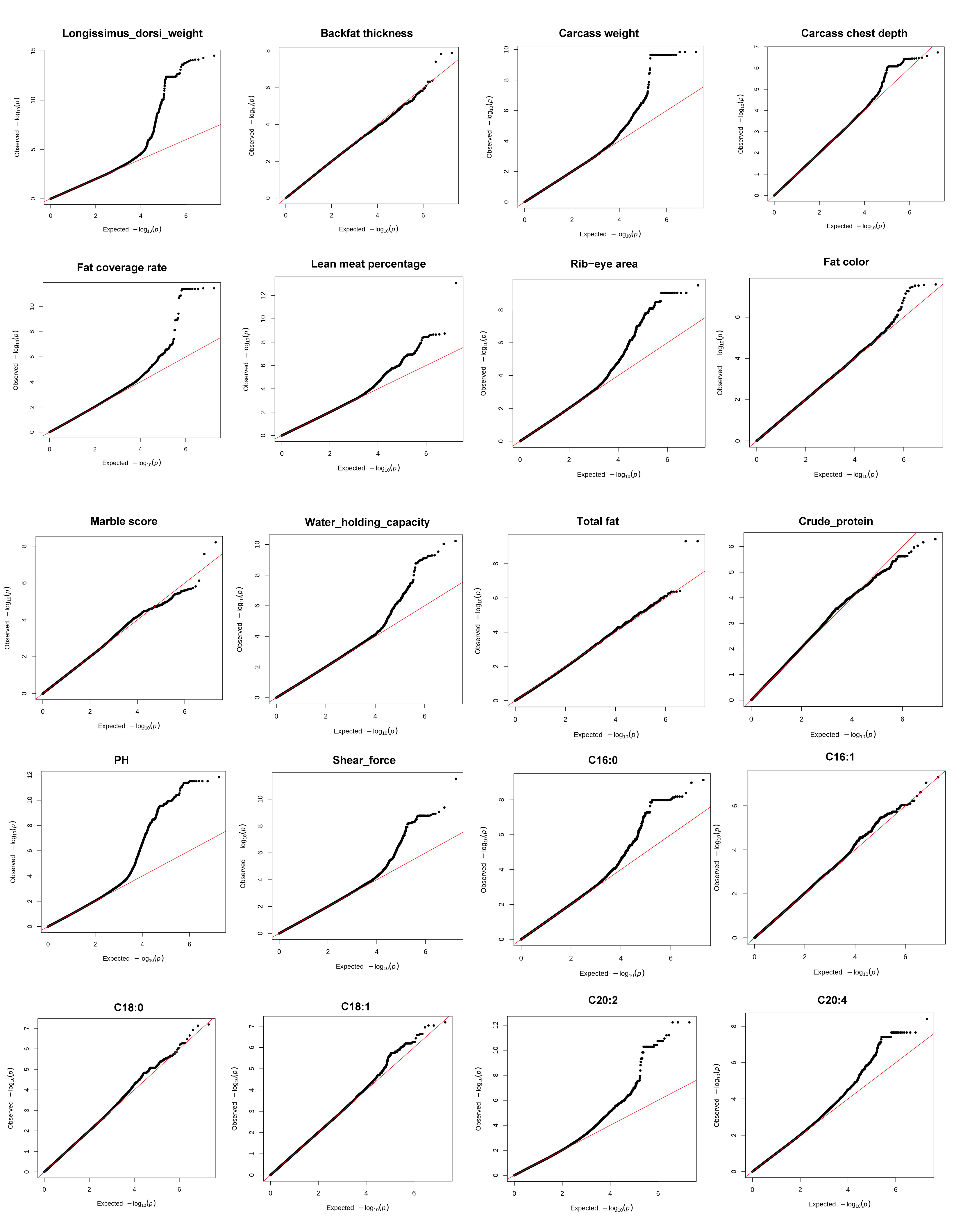 |
| --- |
| **Fig. S1. Quantile–quantile plots for agronomic traits.** Quantile–quantile (Q–Q) plots of *P* values for the 20 agronomic traits. The dashed line indicates the expected distribution under the null hypothesis; departures in the upper tail indicate the presence of association signals. |

| 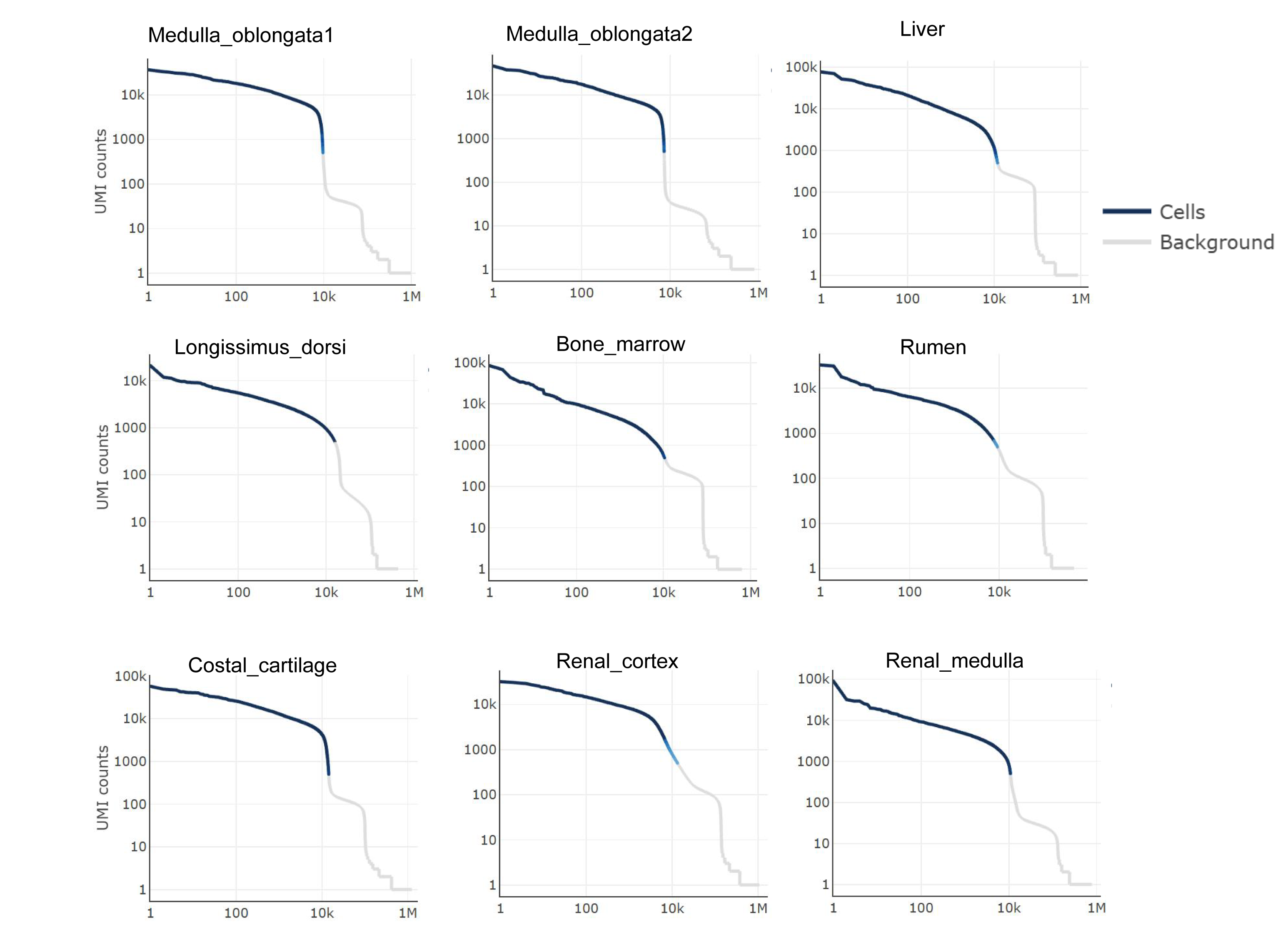 |
| --- |
| **Fig. S2.** Barcode Rank Plot for Cell Calling. Barcode rank plots showing the UMI count distribution for nine samples. The sharp inflection (“knee”) marks the threshold used to distinguish high-quality cells (blue) from background empty droplets (grey). |

| 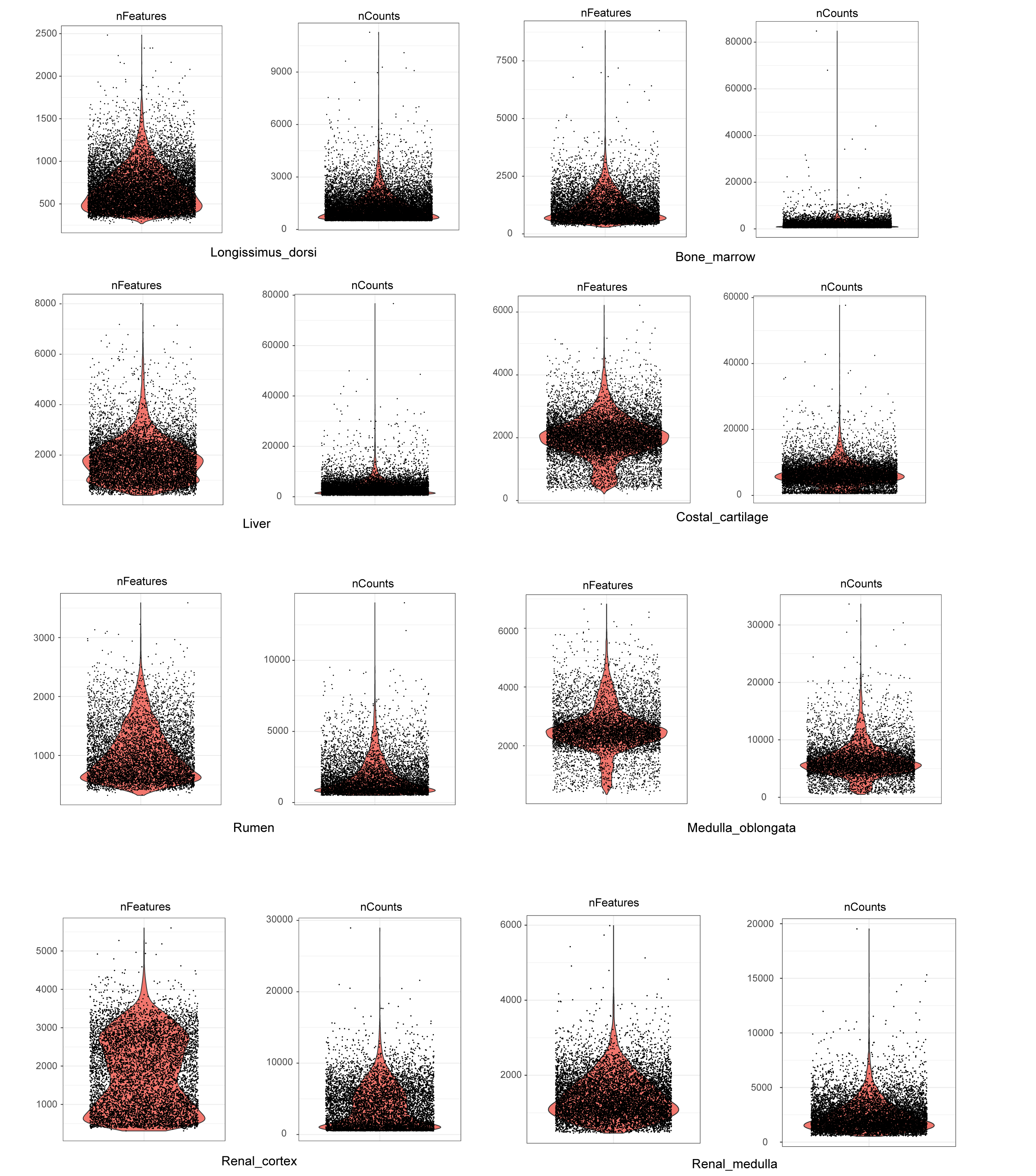 |
| --- |
| Fig. S3. Quality-control metrics across eight tissues. Violin plots showing the number of detected features (nFeatures) and total UMI counts (nCounts) per cell after quality-control filtering in each of the eight tissues. |

| 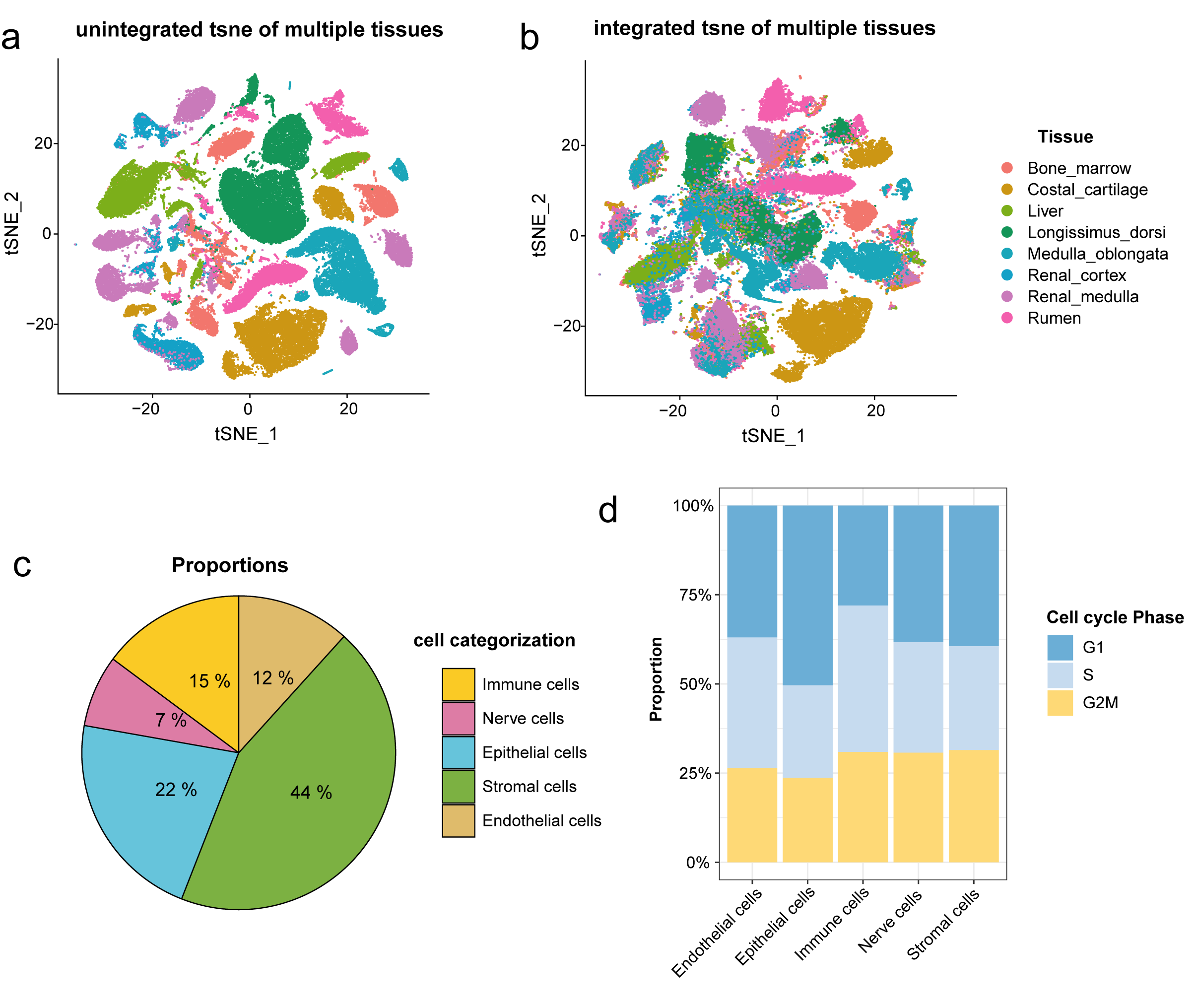 |
| --- |
| Fig. S4 Integration of single-cell atlases and cell-type composition across eight tissues. a, t-SNE visualization of cells from eight tissues before dataset integration. b, t-SNE visualization of the same cells after integration, showing effective reduction of batch effects. c, Pie chart showing the proportional abundance of the five major cellular lineages: immune, neuronal, epithelial, stromal and endothelial cells. d, Bar plot showing the distribution of cell-cycle phases (G1, S and G2/M) within each of the five major cellular lineages. |

| 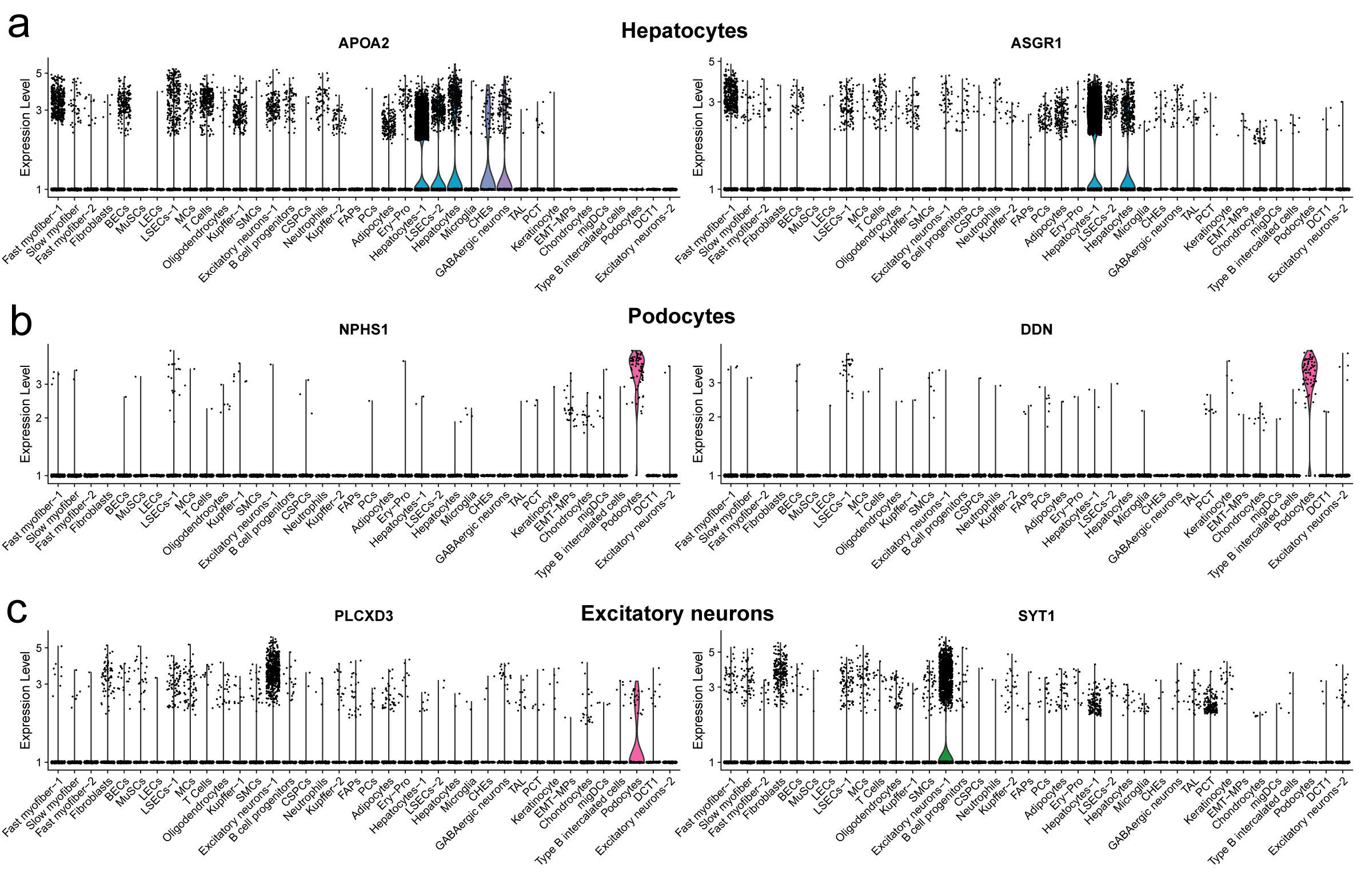 |
| --- |
| Fig. S5. Marker gene expression for selected cell types. Violin plots showing the expression of canonical marker genes used to identify hepatocytes, podocytes and excitatory neurons. |

| **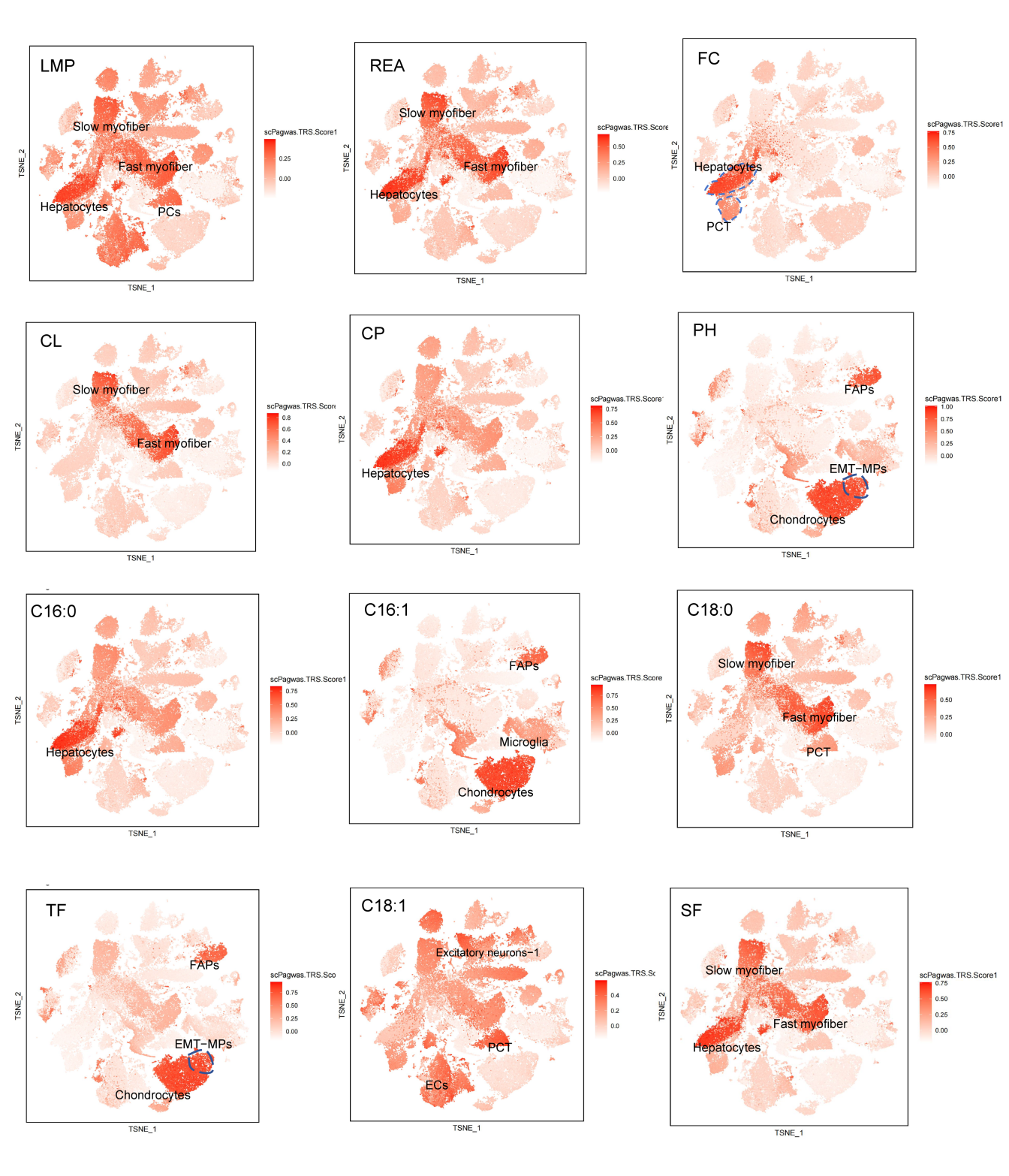** |
| --- |
| Fig. S6. Cell-level trait relevance scores for agronomic traits. Dimensionality reduction visualization of trait relevance scores (TRS) for 12 agronomic traits. Annotated cell types represent those showing significant associations with specific traits. Colour intensity reflects TRS, with darker colours indicating higher scores. |

| 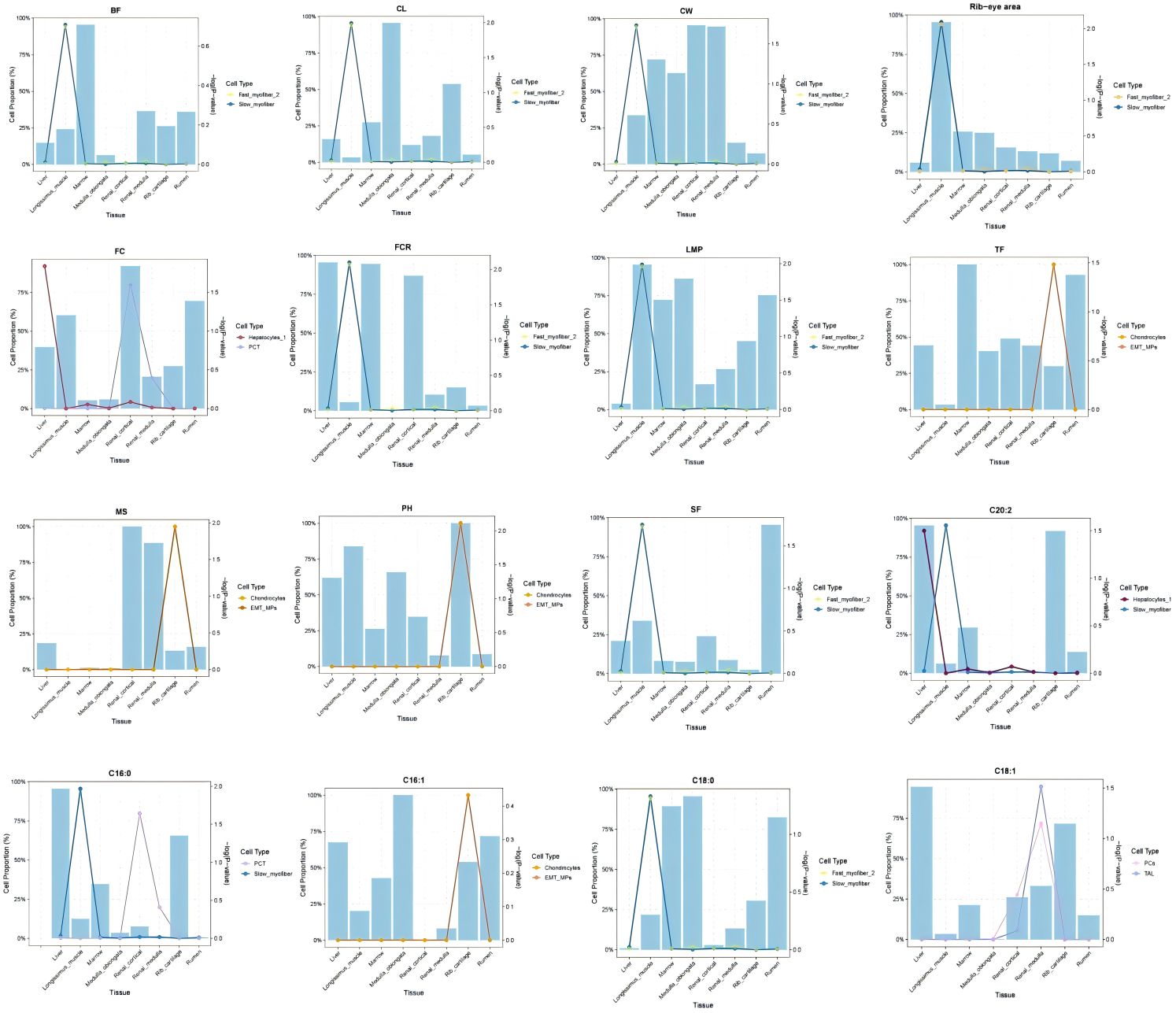 |
| --- |
| Fig. S7. Tissue distribution of top trait-associated cell types. Bar plots showing the proportional abundance (left y axis) of the top two trait-associated cell types across eight tissues. Bar height (right y axis) indicates the strength of trait–tissue enrichment based on MAGMA analysis. This figure shows results for the remaining 14 agronomic traits, complementing the six traits presented in the main figure. |

| 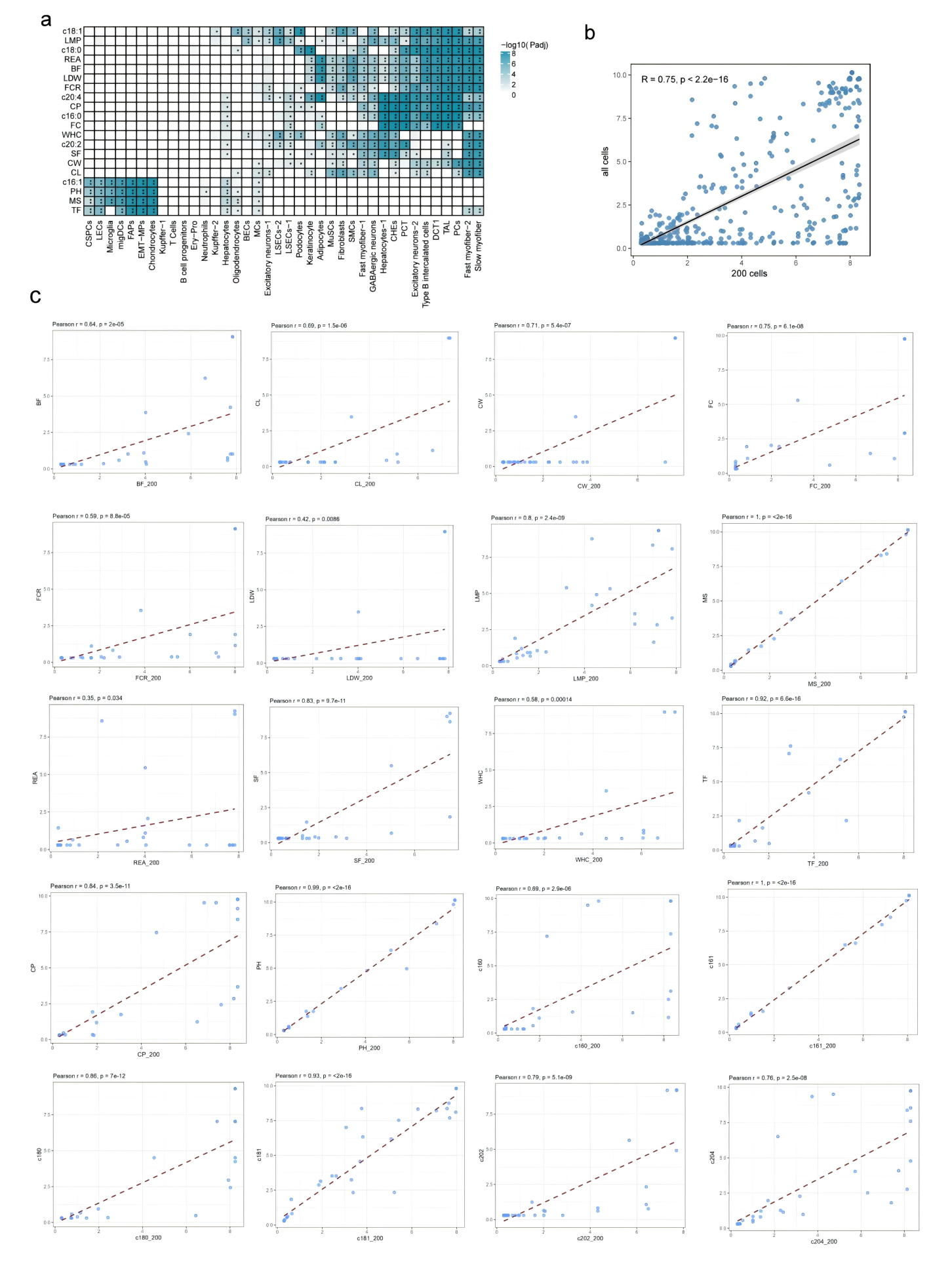 |
| --- |
| Fig. S8. Robustness of cell–trait associations under random subsampling. a, Heat map showing the significance of associations between cell types and agronomic traits when 200 cells are randomly sampled per cell type. **P* < 0.05, ***P* < 0.01. b, Correlation between association results obtained from the subsampled dataset in a (200 cells per type) and the full dataset. Pearson *r* and *P* value are indicated. c, Trait-wise correlation of association results for all 20 agronomic traits between the subsampled (200 cells per type) and full datasets. |

| 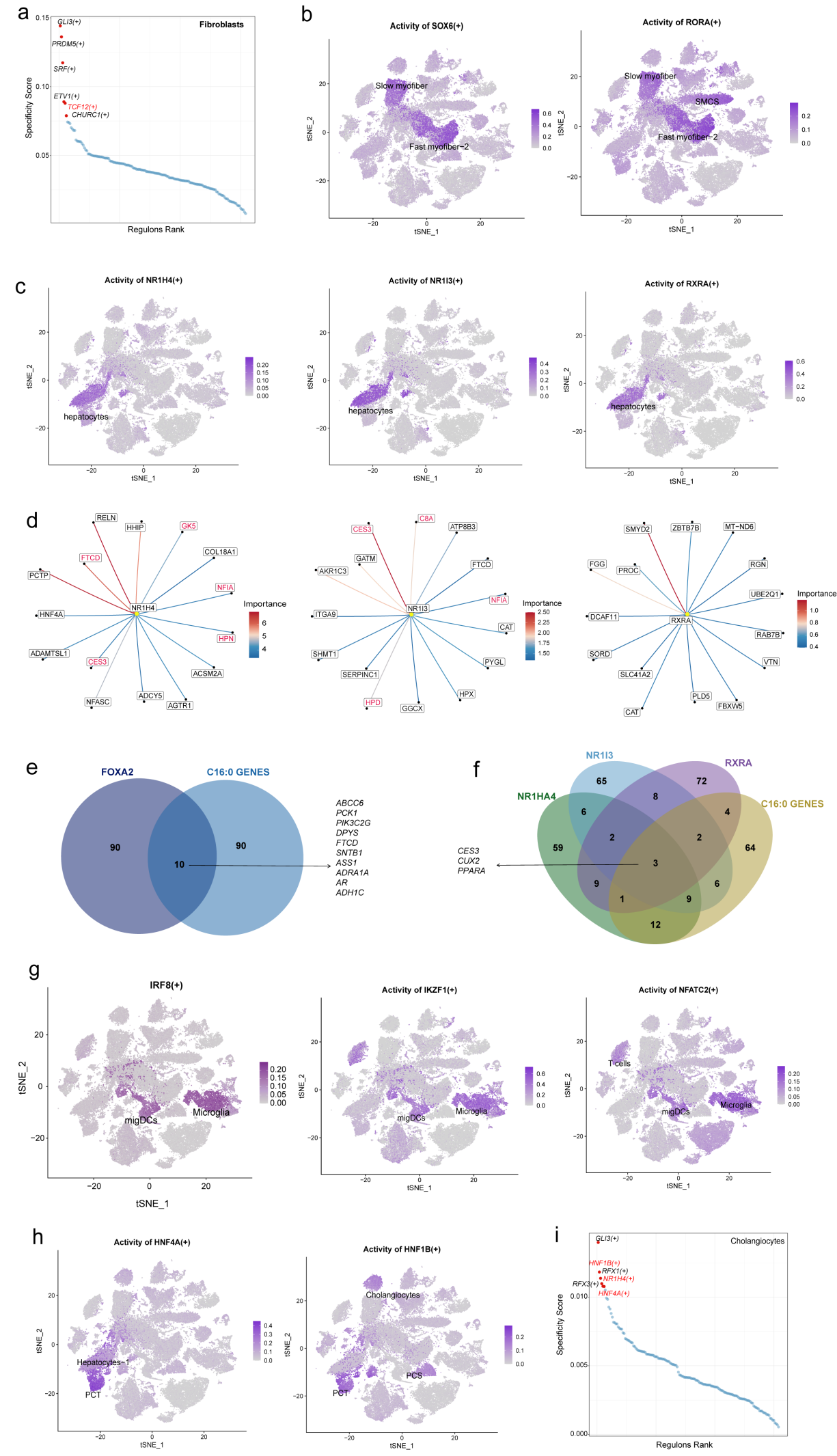 |
| --- |
| Fig. S9. Transcription factor regulatory activity across cell types. a, Transcription factors (TFs) specificity scores in fibroblasts. Fibroblast-enriched TFs are highlighted in red. b, AUCell scores for SOX6 (left) and RORA (right) regulon activity across all cell types. c, Regulon activity of hepatocyte-associated TFs. d, Top 15 target genes of the TFs shown in **c**, ranked by regulon importance. Genes shared with C16:0-associated genes are highlighted in red. e, Venn diagram showing the overlap between FOXA2 target genes and C16:0-associated genes; arrows indicate the shared genes. f, Venn diagram showing the overlap between target genes of the TFs in (c) and C16:0-associated genes; arrows indicate the shared genes. g, AUCell scores for microglia-associated TF regulons across all cell types. h, t-SNE visualization of AUCell scores for HNF4A and HNF1B, illustrating their similar activity patterns in kidney and liver cell populations. i, TFs specificity scores in cholangiocytes. Cholangiocyte-enriched TFs are highlighted in red. |

| 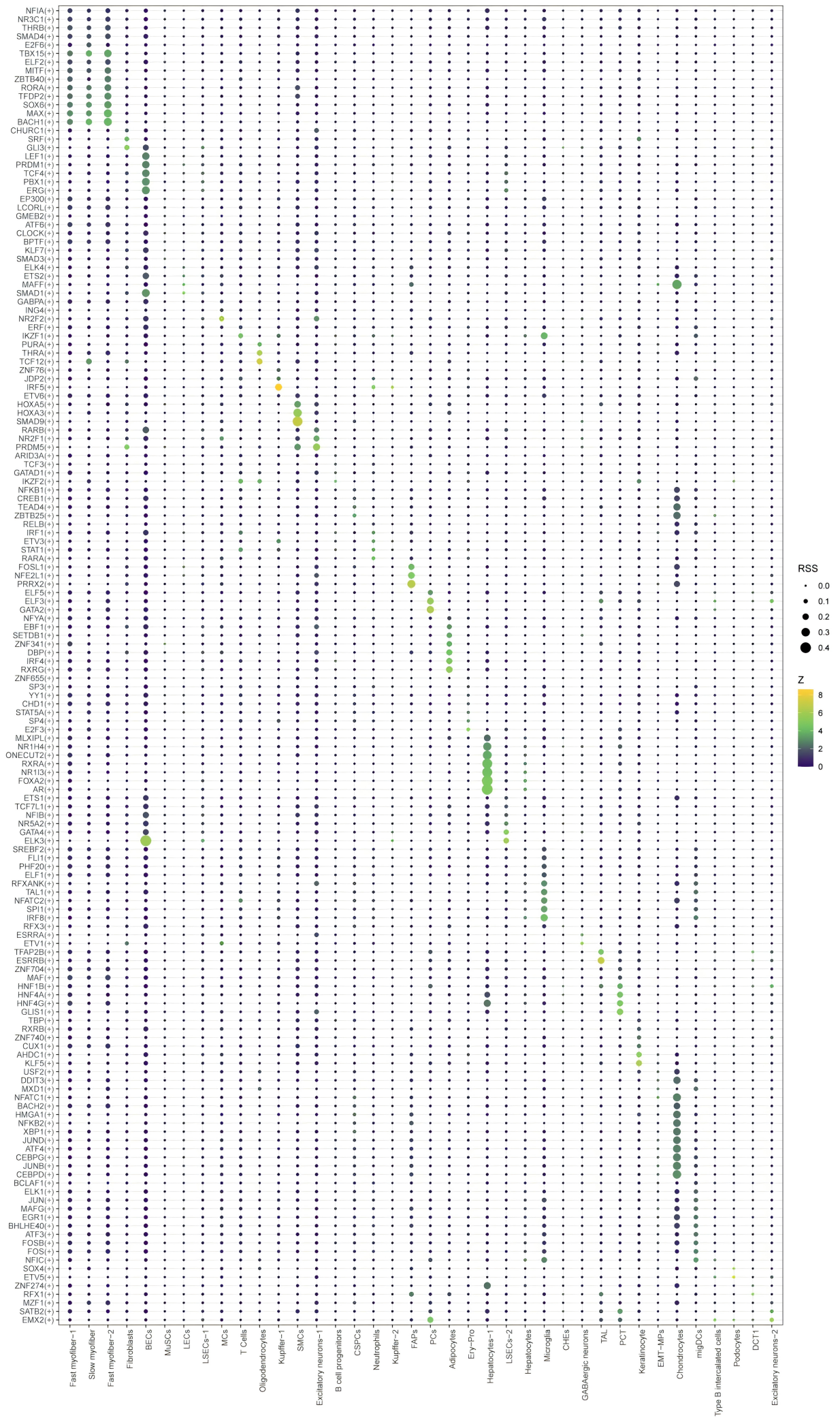 |
| --- |
| Fig. S10. Transcription factor specificity across cell types. Bubble plot showing transcription factor specificity scores (RSS) and corresponding z-scores across all cell types. |
